# Compatibility between snails and schistosomes: insights from new genetic resources, comparative genomics, and genetic mapping

**DOI:** 10.1038/s42003-022-03844-5

**Published:** 2022-09-09

**Authors:** Lijing Bu, Daibin Zhong, Lijun Lu, Eric S. Loker, Guiyun Yan, Si-Ming Zhang

**Affiliations:** 1 Center for Evolutionary and Theoretical Immunology, Department of Biology, University of New Mexico, Albuquerque, NM 87131 USA; 2 Program in Public Health, College of Health Sciences, University of California, Irvine, CA 92697 USA

**Keywords:** Comparative genomics, Genetic linkage study, Sequence annotation, Genetic markers, DNA sequencing

## Abstract

The freshwater snail *Biomphalaria glabrata* is an important intermediate host of the parasite *Schistosoma mansoni* that causes human intestinal schistosomiasis. To better understand vector snail biology and help advance innovative snail control strategies, we have developed a new snail model consisting of two homozygous *B. glabrata* lines (iM line and iBS90) with sharply contrasting schistosome-resistance phenotypes. We produced and compared high-quality genome sequences for iM line and iBS90 which were assembled from 255 (N50 = 22.7 Mb) and 346 (N50 = 19.4 Mb) scaffolds, respectively. Using F2 offspring bred from the two lines and the newly generated iM line genome, we constructed 18 linkage groups (representing the 18 haploid chromosomes) covering 96% of the genome and identified three new QTLs (quantitative trait loci), two involved in snail resistance/susceptibility and one relating to body pigmentation. This study provides excellent genomic resources for unveiling complex vector snail biology, reveals genomic difference between resistant and susceptible lines, and offers novel insights into genetic mechanism of the compatibility between snail and schistosome.

## Abstract (web summary)

Using newly developed genetic resources, comparative genomics, and genetic mapping, this study provides novel insights into schistosome resistance in the snail *Biomphalaria glabrata*, an important intermediate host of *Schistosoma mansoni*, a causative agent of human schistosomiasis.

## Introduction

Schistosomiasis, caused by digenetic trematodes of the blood fluke genus *Schistosoma*, is one of world’s great neglected tropical diseases, and currently afflicts 237 million people in 78 countries^1^. There is no effective vaccine against schistosomes. Current treatment relies almost exclusively on Praziquantel (PZQ), a drug that has been used for more than 40 years^2^. PZQ-based control programs are not likely to be sufficient to achieve the ultimate goal of disease control and elimination because PZQ-treated patients, especially children, often rapidly reacquire infection^3–5^. Snail control, alone or in combination with other strategies, has been proven to be the most effective means in reducing schistosomiasis transmission in endemic areas^6–8^. Strategies currently in use for snail control include biocontrol using competitors or predators, modification of snail habitats, and application of molluscicides. Each approach, however, has been found to have significant limitations, and is not sustainable. Genetics-based vector snail control has long been advocated as a novel biocontrol strategy of schistosomiasis^9,10^ and such a strategy has already shown great promise in control of insect vector-transmitted diseases^11,12^, but not in schistosome vector snails. Studies of the vector snail *Biomphalaria tenagophila* in Brazil^13,14^ provide supportive field evidence for the validity of the approach, though the underlying mechanism for resistance remains to be explored in *B. tenagophila*. To accomplish the exciting goal of schistosomiasis control, a critical understanding of vector snail biology, particularly genetic mechanisms underlying snail resistance to schistosomes, is required^15,16^.

*Biomphalaria glabrata* – *Schistosoma mansoni* has been applied for studies of snail-schistosome interactions for more than half a century^17–20^. The Neotropical species *B. glabrata* is an important intermediate host of the parasite *S. mansoni*, a causative agent of human intestinal schistosomiasis. Two *B. glabrata* strains, M line and BS90, have been most used for research and supplied by NIH-funded Schistosomiasis Resource Center of Biomedical Research Institute (BRI) for world-wide research (www.afbr-bri.org/schistosomiasis)^21^. In general, albino M line is susceptible to *S. mansoni*, whereas pigmented BS90 is resistant to the parasites. M line was selected from early crosses between albino Brazilian strain and pigmented Puerto Rican snails in 1950s^17^. The pigmented BS90, also referred to frequently as the Salvador strain, was isolated from field populations in 1960s in Salvador, Brazil, which were found to be resistant to infection with both local and allopatric *S. mansoni*^22^. By using the two strains, most of the past work has focused on immunological responses of *B. glabrata* to digenetic trematodes, particularly *S. mansoni*^18–20^. Only a few genetics studies concerned with genetic mapping of schistosome resistance in *B. glabrata* have been reported. Knight et al.^23^ identified two schistosome-resistant loci (OPM-04 and OPZ-11) using adult F2 offspring produced from crosses between non-inbred M line and BS90 *B. glabrata* snails. More recently, three new resistant loci, GRC, RADres, and PTC2, have been uncovered from juvenile snails of Guadeloupe and 13-16 R1 strains of *B. glabrata*^24–26^. Although these efforts have significantly improved our knowledge of snail resistance in general, fundamental genetic mechanisms underlying snail resistance to schistosomes remain elusive. For example, all the above-mentioned resistance loci have been found in different genomic regions. Clearly, further investigations using advanced genetic and genomic resources and more powerful genotyping technologies are needed.

We have developed a new snail model consisting of two homozygous lines with sharply contrasting phenotypes of schistosome-resistance, namely iM line and iBS90, which were derived from the M line and BS90 snails as described. To empower this genetic resource, we have sequenced, assembled, and annotated the genomes of the two lines. The quality of the two genomes generated from the two lines representing two distinct phenotypes (resistance and susceptibility) has been significantly improved compared to the first pioneering reference genome of *B. glabrata* reported^27^. To exploit these unique genetic and genomic resources for better understanding genetic mechanisms of snail resistance to schistosomes, we produced F2 segregating population using the two lines as parents, constructed a high-quality linkage map, and identified three novel quantitative trait loci (QTLs) involved in snail resistance and pigmentation using double digest restriction-site associated DNA sequencing (ddRADseq)^28^. Taken together, our findings (two homozygous lines, two genome sequences, one linkage map, and three QTLs) presented in this paper provide valuable genetic and genomic resources for better understanding complex biology of vector snails and offers insights into genetic basis underpinning snail resistance or susceptibility to schistosomes.

## Results

### Generation of two new *B. glabrata* lines

Here we report a new snail model that consists of two new lines, namely iM line and iBS90, which were selected from M line and BS90, respectively (for details see “Methods”; Supplementary Fig. 1a). The two lines possess two important properties, homozygosity of their genomes and sharply contrasting schistosome-resistance phenotypes. K-mer analysis^29^ suggests the iM line (81 consecutive generations of selfing) and iBS90 (39 consecutive generations of selfing) are homozygous (Supplementary Fig. 1b). By the criterion of producing cercariae after exposure to *Schistosoma mansoni* miracidia or not, it has been repeatedly confirmed that iM line is fully susceptible to *S. mansoni* (produce cercariae) whereas iBS90 is completely resistant to the parasite (no cercariae produced). Snails of the iM line are albinos whereas iBS90 snails have pigmented bodies and eyes.

### Sequencing, assembly, and annotation of the two lines

For each homozygous line, two individual snails were used for whole genome sequencing, one for Illumina, and the other for PacBio sequencing (Table 1). The assembled genome size is 871 for iM line (255 scaffolds; N50 = 22.7 Mb) and 885 Mb for iBS90 (346 scaffolds; N50 = 19.4 Mb) (Table 1). Benchmarking universal single-copy orthologue (BUSCO) analysis shows that the calculated completeness of the assembled genome for both iM line and iBS90 is 96% based on a Metazoa dataset of 954 core genes^30^. The overall statistical data indicate that the quality of the iM line genome assembly is better than that of iBS90, so we used the iM line genome as reference for our linkage group analysis and genetic mapping presented in this paper. Table 1 provides detailed information of the two new genome assemblies as well as the first reference genome for *B. glabrata* of the BB02 laboratory strain derived from a Brazilian field isolate^27^.

**Table 1:** Summary statistics of the genome assembly of iM line and iBS90 in comparison with reference genome of BB02 strain.

	iM line	iBS90	BB02
*Genome sequencing and raw data*			
Sequencing platform	Illumina	Illumina	Sanger+454+Illumina
Number of reads	410,797,214	439,992,466	
Mean of read (bp)	150 × 2	150 × 2	
Median of read (bp)	150 × 2	150 × 2	
Total of bases (Mbp)	123,239	131,998	
Coverage	149	154	79
Sequencing platform	PacBio	PacBio	
Number of reads	7,308,853	6,198,071	
Mean of read (bp)	13,712	12,189	
Median of read (bp)	24,502	25,197	
Total of bases (Mbp)	100,217	75,550	
Coverage	120	87	
*Assembly statistics*			
Total length (bp)	870,959,050	885,111,083	916,377,450
Number of scaffolds	255	346	331,400
Mean scaffold length (bp)	3,415,526	2,558,125	2,765
Longest scaffold length (bp)	60,252,455	35,282,478	2,183,814
Shortest scaffold length (bp)	1,308	1,344	200
Scaffold N50	22,698,051	19,395,504	48,059
Scaffold L50	13	18	3,093
GC content	36.14%	36.10%	36.19%
BUSCO	96%	96%	88%
Total length (>=10 Kbp)	870,886,136	885,159,316	644,167,852
Number of contigs (>=10 Kbp)	238	336	11,103

Note: Complete and fragmented BUSCOs found from OrthoDB v10 Metazoa datasets (*N* = 954, https://busco.ezlab.org/list_of_lineages.html). Data of BB02 strain were from Adema et al.^27^.

A total of 35,015 gene models for iM line were predicted using annotated BB02 proteins and RNA-Seq data available in public databases. Among four functional annotation analyses applied, 28,554 genes (81.54% of the 35,015) were annotated from at least one of the four sources: (1) BLASTp against high-quality manually curated Uniports database (15,263 counts for 44% of the total); (2) BLASTn against NCBI non-redundant nucleotide (NT) database (23,781 count for 68% of the total); (3) NCBI non-redundant protein (NR) database (23,673 count for 68% of the total); and (4) domain prediction from InterProScan and IgSF domain models (23,949 count for 68% of the total) (Supplementary Fig. 2a). Using the same approach, a total of 38,516 gene models were predicated in iBS90 (Supplementary Fig. 2b). We found 21,368 ortholog groups between 23,142 iM line genes and 23,318 iBS90 genes (not all genes have ortholog between the two lines). In addition, the iM line and iBS90 genomes contain 42.95% and 55.17% repetitive elements, respectively; more unclassified repeats were also found in iBS90 genome (Supplementary Fig. 3, Supplementary Table 1).

### Structural variation of the genomes between the two lines

**Fig. 1:**
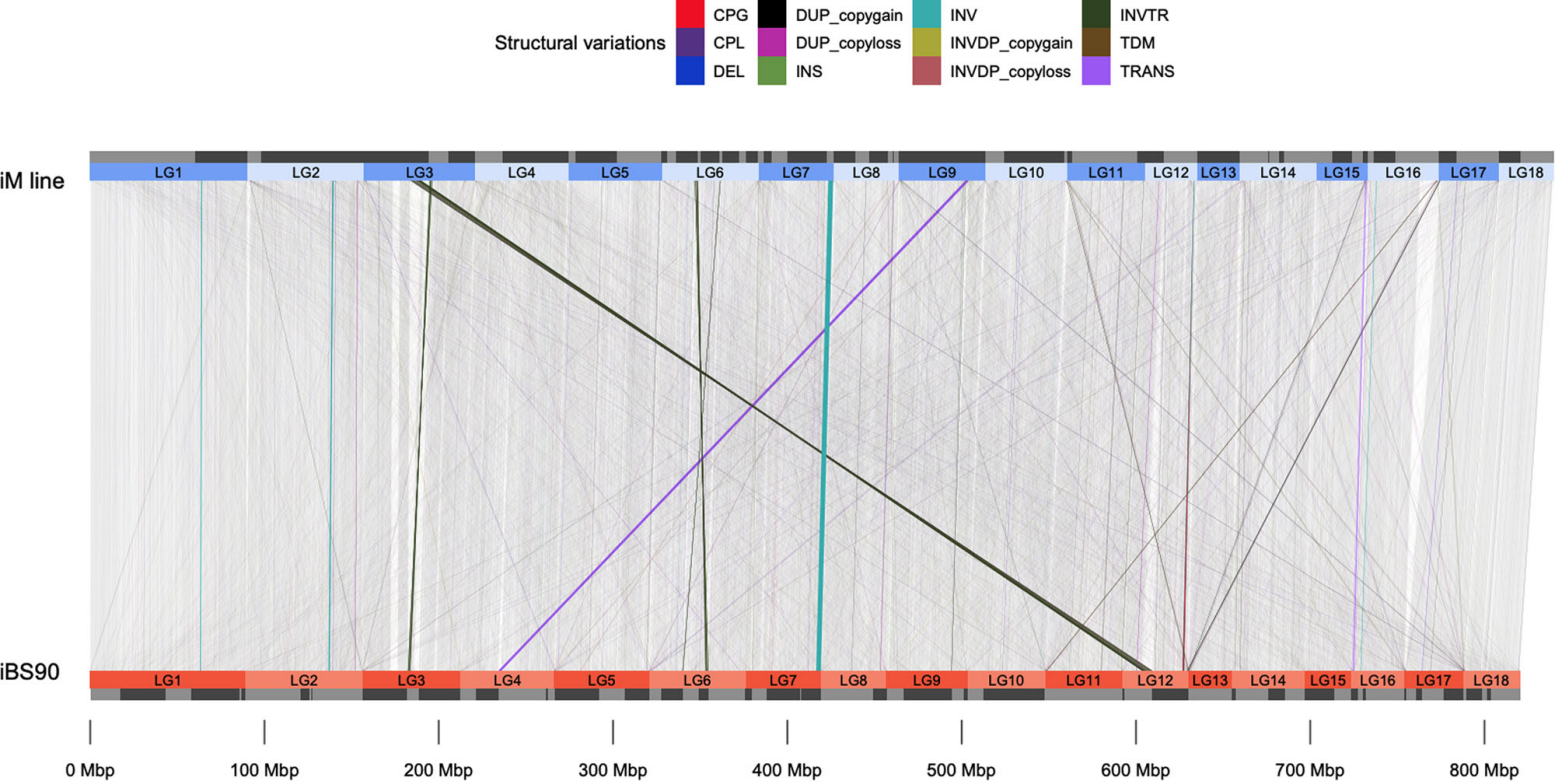
Synteny plot and structural variations (SVs) between assemblies of iM line and iBS90. The numbers of linkage group were assigned based on the sum of lengths of the scaffold on each linkage group. The linkage groups are marked in colored boxes for iM line (blue on top) and iBS90 (orange at bottom). Separated scaffolds inside each linkage group were marked out as gray and black boxes. Synteny blocks between two genomes are laid out in gray blocks in the background. SV types were highlighted in different colors. Cpg: copy gain in query, Cpl: copy loss in query, DEL: deletion in query, DUP: duplicated region, HDR: highly diverged regions, INS: insertion in query, INV: inverted region, INVDP: inverted duplicated region, INVTR: inverted translocated region, NOTAL: un-aligned region, SNP: single nucleotide polymorphism, SYN: syntenic region, TDM: tandem repeat, TRANS: translocated region.

**Fig. 2:**
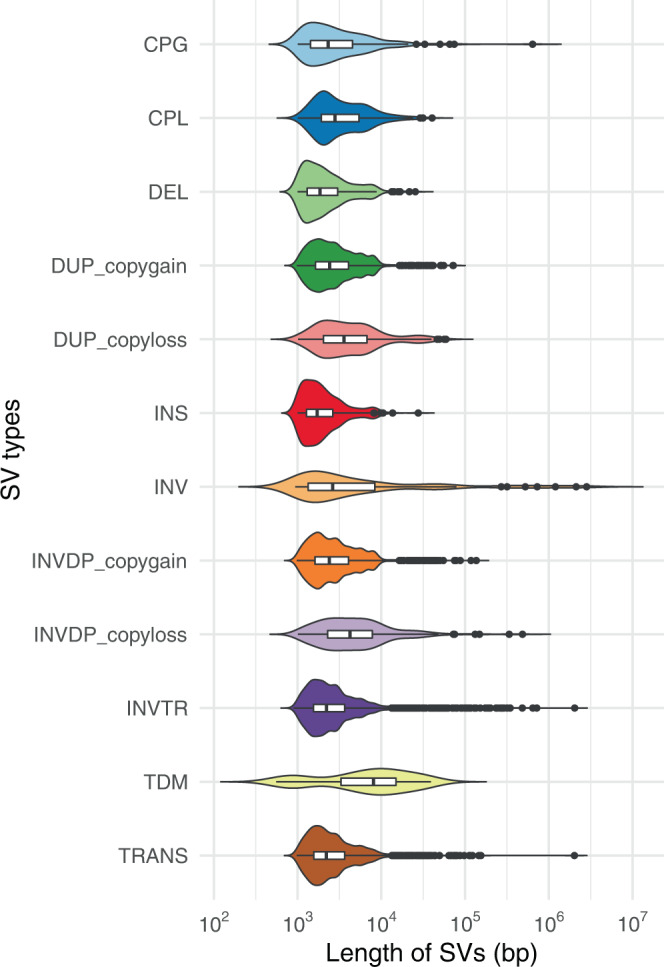
Violin plots of SVs lengths distributions between iM line and iBS90 genome assemblies. CPG: copy gain in query, CPL: copy loss in query, DEL: deletion in query, DUP: duplicated region, INS: insertion in query, INV: Inverted region, INVDP: inverted duplicated region, INVTR: inverted translocated region, SYN: syntenic region, TDM: tandem repeat, TRANS: translocated region.

A large number of small structural variations (SV) was found between the two genomes (Fig. 1). The total size of genomic fragments involved in SV was 50 Mb, with a size ranging from 536 bp to 2.8 Mb (median of 4.6 Kb), which covers 5.8% of the whole genome (Supplementary Table 2, Fig. 1). The first quartile (25th percentile) is 1.5 Kb and the third quartile (75th percentile) is 4 Kb, suggesting overall SVs are small. Large-scale sub-chromosomal level rearrangements were not detected between the two genomes. Detailed breakdown of sizes for each SV type is presented as a violin plot (Fig. 2). The SVs were found in 60 scaffolds, overlapping the exons of 1446 protein-coding genes (Supplementary Data 1). This suggests that differences with respect to the schistosome-resistance trait between the two lines may not result from a large-scale structural variation.

### Phenotypic analysis of F2 progenies bred from the two lines

By using the iM line (albino) and iBS90 line (pigmented) as starting parentals, we generated a F2 segregating population for genetic studies (Supplementary Fig. 1a). Of 869 F2 juvenile snails tested for schistosome-resistance phenotype, 421 were found to be resistant to *S. mansoni* and 448 were susceptible. A Chi-square goodness of fit demonstrated that the ratio of resistance to susceptibility (or vice versa) did not display as ratio of 3:1 (*P* < 0.05). This suggests schistosome resistance in juvenile snails is not likely to be controlled by a single gene (Chi-square value for the homogeneity: *χ*^2^ = 18.78, *P* < 0.01), which is in an agreement with previous studies using classic genetic crossings^31,32^. In addition, body color (pigmented or albino) or eye color (pigmented snails also have black eyes) of the same F2 snails was also recorded, and 674 pigmented and 195 albino F2 progenies were obtained (*χ*^2^_3:1_ = 3.04, *P* = 0.08), indicating a single gene or locus is involved in controlling snail pigmentation (Chi-square value for homogeneity: *χ*^2^ = 28.30, *P* < 0.0001). There was no evident association between albinism/pigmentation and resistance or susceptibility status to *S. mansoni*.

### Construction of a linkage map

From 126 ddRAD (*EcoR* I and *Msp* I) libraries generated, 122 F2 snails and 4 parent snails (2 from either iM line or iBS90), were successfully sequenced using the Illumina HiSeq platform (Supplementary Data 2). The cleaned reads were mapped to iM line and iBS90 genomes, allowing the identification of 90,898 and 92,143 high-quality single nucleotide polymorphisms (SNPs), respectively (Supplementary Fig. 4a, b, c, d). The transitions (C/T and A/G) over transversions (A/C, G/T, A/T, and C/G) ratio was 1.86 in SNPs based on the two genomes. A total of 1482 SNPs (1.63%) for iM line and 1546 (1.68%) for iBS90 were exonic, while the remaining SNPs were found in intronic and intergenic regions. High proportions of shared detection and comparable SNP markers were revealed upon independent assessment of each genome, suggestive of high-quality genome assembly of the two lines. To simplify the study, subsequent analyses of SNPs were performed based on the iM line genome.

**Fig. 3:**
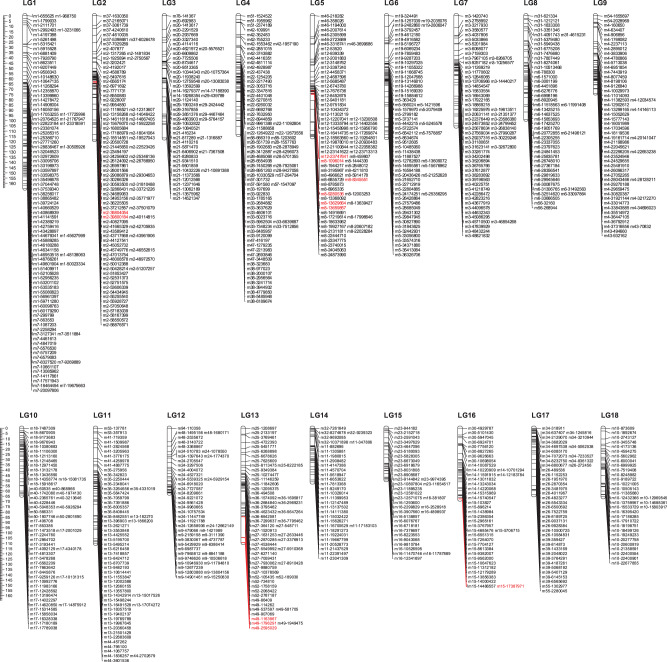
A genetic linkage map based on a F2 offspring bred from iM line and iBS90. The markers were labeled by the scaffold # followed by the physical position of the SNP. Markers in red represent those markers used for validation by Sanger sequencing.

Of the 122 F2 snails, 116 were retained after removing individuals with incomplete or low-quality genotype information. Of 90,898 biallelic SNPs, 17,392 were retained after filtering or removing SNPs with high missing data (>35%), apparent segregation distortions (*P* < 0.001) and genotype error, and loci with high levels of pairwise linkage disequilibrium (LD). After tagging of 17,392 SNPs using HaploView with 3.0 of LOD (logarithm of the odds) and r-squared correlation coefficients (<0.8), and 100 bp of minimum distance between loci, a set of 1146 reliable SNPs was selected for linkage map construction. Of 1146 segregating SNP loci, 996 (~87%) were assembled into the 18 large linkage groups (LGs) with the most numbers of markers (>32 SNP loci) at a LOD score of 9.0 and a maximum recombination frequency of 0.14 (Supplementary Data 3). Among the remained 150 SNP loci, half of them (*n* = 75) are either orphan markers unlinked to other makers or located in linkage groups with markers less than 10, so these 150 SNP loci were excluded from linkage map analysis. The numbers for linkage groups were assigned based on the total length of scaffolds on the linkage group. The markers on the genetic linkage map presented the same sequential order as that presented in the physical map from the iM line reference genome. The final genetic linkage map consisted of 18 LGs, which contain 996 SNP markers and span 1664.7 Kosambi cM with an average distance between markers across the 18 linkage groups of 1.73 cM (Fig. 3). The genetic distance of linkage groups ranged from 49.1 cM (LG15) to 164.7 cM (LG1). The number of SNP markers mapped to each linkage group varied from 32 markers on LG14 to 93 markers on LG1, with an average of 54 SNPs per linkage group. The 18 LGs that represent the 18 haploid chromosomes characteristic of *B. glabrata* (2*n* = 36)^33^, were composed of 64 scaffolds, which cover 96% of the assembled iM line genome (Fig. 3, Supplementary Data 3, 4).

**Fig. 4:**
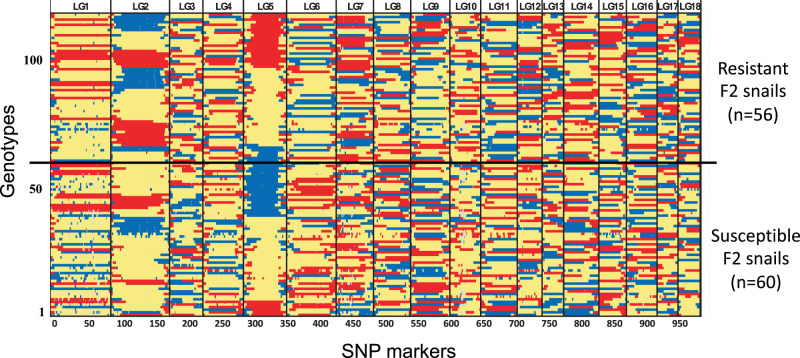
Graphical representation of genotypes on 18 linkage groups. Blue, red, and yellow colors represent alleles that are derived from resistant iBS90, susceptible iM line and heterozygous (from both parents), respectively. The white color indicates missing information. Black vertical lines indicate the boundaries between LGs. The y-axis was sorted by genotypes of QTL regions on LG5 and LG2 to show the proportion of genotypes in resistant group (upper) and susceptible group (lower).

**Fig. 5:**
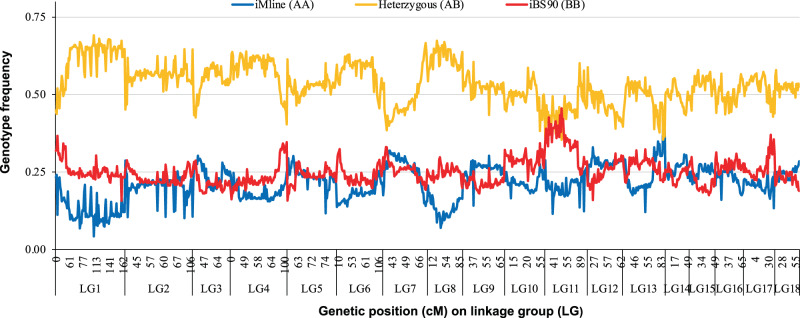
The proportion of contributing alleles from iM line (AA) and iBS90 (BB) for each marker on the 18 linkage groups. Parental allele contributed by iM line and iBS90 is represented in blue and red, respectively, whereas heterozygous contributed by both parents is indicated in yellow.

Given an estimated size of 871 Mb in the genome assembly of iM line (Table 1), the current linkage map is ~0.52 Mb/cM. The recombination blocks were clearly defined in the graphical representation of genotypes (Fig. 4), indicating that the genotypic dataset used for construction of the final linkage map was well-corrected and suitable for linkage mapping^34^. In the pairwise recombination fraction plot, some markers had high recombination frequencies (>0.5), but low LOD scores (Supplementary Fig. 5a). The heatmap of estimated recombination and LOD scores of mapped markers showed a low level of off-diagonal association between marker pairs and consistency of square-like heat blocks across the diagonal line within chromosomes, suggesting this is a good-quality linkage map (Supplementary Fig. 5b)^35^. Of the 996 mapped SNP markers, 121 (12.1%) showed significant (*P* < 0.01) distortion from the 1:2:1 segregation ratio in the F2 population, with heterozygote deficiency (LG11 and LG13) or heterozygote excess (LG1 and LG8) (Fig. 5).

### QTLs conferring schistosome-resistance and body pigmentation

**Fig. 6:**
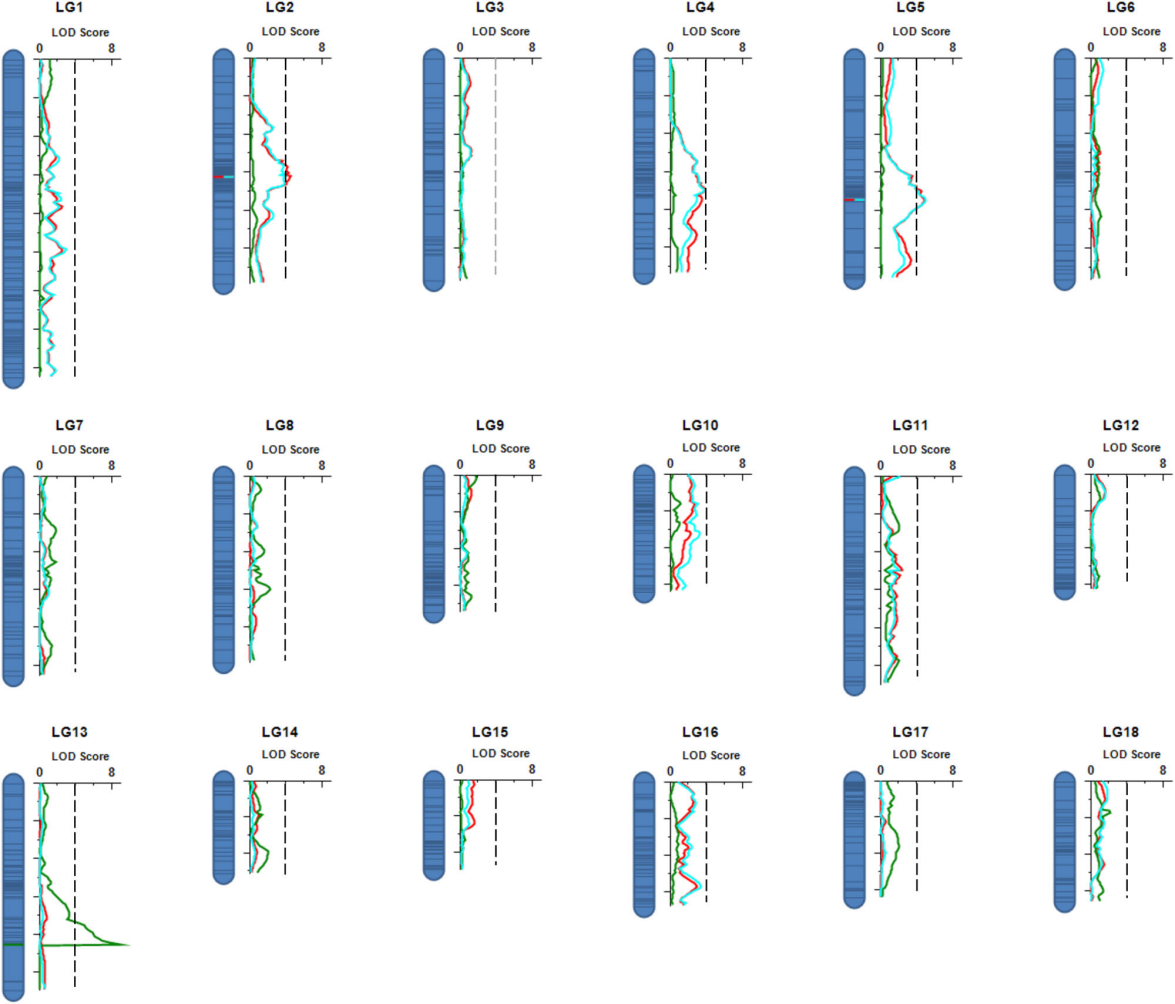
LOD profile of QTLs detected on linkage group 1 (LG1) to LG18 in *B. glabrata*. Two QTLs were detected on LG2 and LG5 for host resistance/susceptibility (in red) and parasite abundance (in blue), while one QTL detected on LG 13 for pigmentation (in green). Gray dot lines show threshold LOD score (LOD = 4) used for detecting QTL.

Using a genome-wide LOD significant threshold of 4.0 and permutation test^36^, two QTLs (qRS-2.1 and qRS-5.1) associated with schistosome resistance/susceptibility were detected on two LGs or chromosomes (LG2 and LG5) using simple interval mapping (SIM) (Fig. 6, Table 2). Relatively high LOD scores were noted in LG4 and LG10, but not selected because they did not meet the criterion (i.e., LOD > 4). The peak of largest effect (qRS-5.1) was located between markers m8-12003253 and m8-13368092, with a genetic distance of 0.50 cM or physical distance of ~1.36 Mb. This QTL had an additive effect of 0.30 for increased resistance to parasite infections when an individual harbored the allele from a resistant parent. The second QTL (qRS-2.1) is located between the markers m2-36225505 and m2-37212557 and has a genetic distance of 1.43 cM or physical distance of ~0.99 Mb. This QTL had a dominance effect of 0.41 for reduced resistance to parasite infections when an individual harbored the allele from a susceptible parent. In addition to using lack of cercarial shedding as a resistance phenotype as just described, we also examined the abundance of parasite DNAs/reads present in the snail body as an indication of schistosome resistance. For a given snail, the more schistosome reads counted in its DNA sample, the more susceptible to schistosomes the snail was considered to be. Therefore, reads that were unmapped to the snail genome but mapped to the *S. mansoni* genome^37^ were used as an indication for abundance of parasites present in a snail (Supplementary Fig. 6, Supplementary Data 2). Remarkably, two QTLs (qsm-2.1 and qsm-5.1) revealed based on the number of schistosome reads are consistent with the patterns observed by the resistance phenotype (cercariae shedding) for both QTL regions (qRS-2.1 and qRS-5.1), except for varying confidence intervals, supportive of the high reliability of the two QTLs identified (Fig. 6; Table 2). The size of the two QTLs (qRS-2.1 and qRS-5.1) covers an interval of 0.5–1.43 cM in genetic distance or 0.99–1.36 Mb in physical distance, while the 95% confidence intervals of QTLs span large chromosomal regions ranging from 2 to 8 cM genetic distance or 4.03–21.36 Mb physical distance. Interestingly, both QTLs locate in centromeric and pericentromeric regions inferred from the genetic map as corresponding to large, low or zero recombination regions^38^, suggesting centromeric/pericentromeric regions may play a role in schistosome resistance/susceptibility in *B. glabrata*.

**Table 2:** Quantitative trait loci (QTL) of schistosome-resistance and pigmentation detected by simple interval mapping (SIM).

Trait	QTL	LG	Position (95% CI) (cM)	QTL peak Left Marker	QTL peak Right Marker	LOD	PVE (%)	Add	Dom	QTL peak size (95% CI size) (cM)	QTL peak size (95% CI size) (MB)
Resistance	qRS-2.1	2	62 (61.5, 66.5)	m2-36225505	m2-37212557	4.47	12.73	0.06	0.41	1.43 (5.0)	0.99 (7.95)
	qRS-5.1	5	74 (72.5, 79.5)	m8-12003253	m8-13368092	4.79	13.27	0.30	0.03	0.50 (7.0)	1.36 (6.13)
Parasite density	qsm-2.1	2	62 (61.5, 63.5)	m2-36225505	m2-37212557	4.02	11.60	0.03	0.20	1.43 (2.0)	0.99 (4.03)
	qsm-5.1	5	74 (71.5, 79.5)	m8-12003253	m8-13368092	4.92	13.70	0.16	−0.01	0.50 (8.0)	1.36 (21.36)
Pigmentation	qBC-13.1	13	85 (84.5, 85.5)	m49-1163967	m49-1796291	8.92	63.20	0.34	−0.18	0.24 (1.0)	0.63 (0.89)

*LG* linkage group, *cM* CentiMorgan, *LOD* logarithm of the odds, *CI* confidence interval, *PVE* percentage of total phenotypic variance explained by the QTL, *Add* additive effect, *Dom* dominance effect.

Regarding body pigmentation, one major QTL (qBC-13.1) was detected. This QTL was found on LG13 and has very high LOD score (8.92) with an additive effect value of 0.34 (Fig. 6; Table 2). No minor QTLs (PVE < 10%) was detected for snail pigmentation.

To validate the association between the significant SNPs and snail phenotypes, 94 F2 snails (randomly selected from those used for ddRADseq) and 48 F2 snails (not used for ddRADseq) were analyzed by Sanger bi-directional sequencing. Eleven markers from the three loci (qRS-2.1, qRS-5.1, and qBC-13.1) and one previously reported (i.e., OPM-04, GenBank accession: AF078109^23^), were successfully genotyped by Sanger sequencing. The odds ratio (OR, a measure of strength of association) of the 12 markers ranged from 2.27 to 11.12 (*P* < 0.01) for technical validation and 3.39 to 8.10 (*P* < 0.05) for biological validation (Supplementary Tables 3, 4). The independent analysis validates these QTL loci identified.

### Genes presented in the QTLs

Based on the genome annotation of iM line presented in this study, 1,348 genes were found in the two loci within the 95% confidence intervals for the QTL regions (a region of ~7.95 Mb on LG2 and ~21.36 Mb on LG5). Among the 1348 genes, a total of 328 were observed to be associated with at least one significantly differential SNP (a total 1315 SNPs) (Supplementary Data 5, 6). Of these 328 genes, 97, 222, and 9 genes were from qRS-2.1, qRS-5.1, and qBC-13.1, respectively. For the 97 genes from qRS-2.1 (448 SNPs), six genes (e.g., epithelial membrane protein and vacuolar protein 8) that possess non-synonymous coding mutations (Supplementary Table 5) and several genes (e.g., neuronal acetylcholine receptor and phosphoribosyl pyrophosphate synthase-associated protein) were found with mutations within putative regulatory regions (intron, upstream and downstream protein-coding sequences) (Supplementary Table 6). For the 222 genes (804 SNPs) located in qRS-5.1, eight had non-synonymous coding changes (e.g., receptor-type tyrosine-protein and serine/threonine-protein kinase, Supplementary Table 5) and several genes (e.g., ferric-chelate reductase, peroxidasin, and orexin receptor) had mutations within the regulatory regions (Supplementary Table 6). Gene ontology (GO) analyses for both resistance QTLs showed that those genes were mainly enriched in specific processes, including the highest enrichments of “binding” term for molecular function and “cellar process” term for biological process. In the cellular component, the highest enrichment was found in membrane proteins (Supplementary Fig. 7a, b).

With respect to the pigmentation locus qBC-13.1, all 9 genes associated with a total of 63 SNPs, including a neuropeptides capa receptor, displayed a single Gln239Lys non-synonymous mutation and several genes (e.g., ATP-dependent DNA/RNA helicase DHX36 and ADAM family mig-17) were found to possess mutations in regulatory regions, but not coding regions. A large deletion was not observed in the coding regions of the genes. The highly enriched genes in pigmentation locus are involved in catalytic activity and cellular processes (Supplementary Fig. 7c).

## Discussion

The draft *B. glabrata* genome was reported in 2017^27^ and provides an invaluable resource for gene discovery and data mining. This pioneering reference genome sequence was generated from many snails using multiple technologies available at the time, including Sanger-, 454-, and Illumina-sequencing. As such, the sequence assembly was highly fragmented (N50 = 48.1 Kb; number of scaffolds = 331.4 K) (Table 1). Recently, three significantly improved and assembled, but not annotated, genome sequences of the *B. glabrata* 13-16-R1 strain that were used for genetic mapping^26^ were released to GenBank (Assembly number: ASM1452496, ASM1452502, and ASM1452495). The numbers of scaffolds for the three genomes are 927, 2718, and 3492, respectively, and corresponding sizes of assembled genomes are 852, 811, and 768 Mb. The two genomes we report here were built on 255 (iM line) and 346 (iBS90) scaffolds, each derived from two homozygous individuals. Using homozygous lines circumvents drawbacks associated with the use of heterozygous strains, which impair production of high-quality genomes^39^. Although extreme inbreeding may result in homozygous representation of deleterious alleles, which may lead to undesirable traits and affect fitness, our homozygous lines maintain contrasting phenotypes with respect to schistosome resistance, preserving the essential trait for our studies of snail-schistosome interactions. Therefore, the two new lines with sharply contrasting vector competence phenotypes and high-quality genome sequences provide valuable genetic and genomic resources for studies of vector snail biology and host-parasite interactions. The unique genetic resources (iM line and iBS90) along with their genome sequences and the data used for linkage and QTL mapping will be provided or released to the scientific community as soon as this work is published. As the quality of the susceptible iM line genome is better than that of the resistant iBS90 genome or of other *B. glabrata* genomes (Table 1^26,27^), the iM line genome can serve as an improved reference genome for *B. glabrata*. An additional step we discuss below to make the iM line even more valuable as a reference genome for schistosomiasis studies, is generation of chromosome-level assemblies.

ddRADseq, a powerful tool for large-scale, high-throughput genotyping, has been used effectively to develop linkage maps and for genetic mapping in many non-model genetic organisms^40–42^. We employed ddRADseq to identify genome-wide SNP markers and then constructed a highly saturated genetic linkage map using an F2 segregating population derived from two homozygous snail parents. The 18 linkage groups, representing the 18 haploid chromosomes (2*n* = 36 in *B. glabrata*) were, in general, consistent with the 18 large LGs reported previously^43^ although a few scaffolds from a given LG are not exactly matched (Table 3, Supplementary Table 7). Different from the previous version consisting of 24 LGs and 56% genome coverage^43^, our version built on the iM genome contained 18 LGs and covers 96% of the *B. glabrata* genome.

**Table 3:** List of some key loci/genes in the 18 linkage groups.

Linkage group	G3-LG	Genetic distance (cM)	Physical distance (MB)	Key loci/genes	References
LG1	I	164.7	90.5	infPhox, Ferritin, duox1, duox303, duox584, Matrilin1, BgGRN, ITGA3, prx621	^101–103^
LG2	II	118.3	66.7	BgCREB, Coagulation factor XI, qRS-2.1, BgRelish	^101,104^
LG3	III	116.7	63.6	P450IIf2, P450If2, P450-2C3, BgRel, GalectinIIa	^101,104–106^
LG4	IV	113.4	55.4	PGRP-SA, PGRP, PGRP-LA1, PGRP-LA2	^107^
LG5	V	115.6	53.6	prx1, TPx, gpx65, HSP40, qRS-5.1, gpx97, GalectinIIIa, FReM, Cystatin	^101,102,105,108^, this study
LG6	IX	116.3	53.6	GRC, cat42, BgTLR, spondin-1, bmplys1, bmplys2, bmplys3, SOD1, prx4, aif, nox2, schistosomin	^24,25,101,102,109–113^
LG7	VI	105.3	49.4	LBP, phox22, GNBP, β-1, 3-glucanase, P4503A24, Inter alpha-trypsinin hibitor	^101,102,105,107^
LG8	VIII	98.9	47.2	hcl-1, hcl-2, BgTEP, bmplys 8-12, 15, 17-23, P4503A56	^105,112,114,115^
LG9	XI	73.9	44.6	fibrillin-1, importin-7	This study
LG10	VII	63.7	43.8	BgSTAT1, BgSTAT2, RADres	^25,104^
LG11	XVIII	109.8	43.4	AIG-a, -c, -e, -h, -i, -j, -k	^75^
LG12	XIII	62.0	41.0	Theromacin, gpx2404, Matrilin, MIF2	^101,102^
LG13	X	109.3	37.1	p63, p53, HSP20, FREP1, FREP12, FREP3, FREP14, FREP11, FREP2, FREP6, FREP13, FREP7, FREP5, FREP10, FREP4, qBC-13.1	^101,108,116–120^, this study
LG14	XV	51.2	34.3	FnBPA, acetylcholinesterase, BgTLR3, BgTLR13, catalase	This study
LG15	XIV	49.1	31.5	Metalloproteinase, AIG-f, bmplys13	^75,101,112^
LG16	XII	67.6	29.7	Dermatopontin, MIF1, bmplys4-7, HSP70B2, PTC2, AIG-g, OPM-04	^23,26,75,102,105,112,121^
LG17	XVI	63.5	29.1	arg1386, arginase	^102,106^
LG18	XVII	65.3	24.5	β-IAP, SPI, HSP70	^101,105^

G3-LG: LGs reported by Tennessen et al.^43^. Please also see Supplementary Table 11 for the comparison between the two linkage maps.

We observed a small portion (12.1%) of SNP markers that did not follow the Mendelian segregation ratio (1:2:1) in the F2 population. Most of those markers located on LG1 and LG8, showed a significant excess of heterozygosity. The exact reason leading to heterozygote excess is not known, but this phenomenon is commonly found in animal and plant species and believed to act as an important evolutionary force^44^. The distorted markers were generally clustered and displayed the same skew direction, suggesting segregation distortion loci under selection on linked marker loci^45^. Consequently, zygotic selection, such as the differentiation of zygote viability or heterosis, may be the genetic mechanism for the observed segregation distortion^46^. In addition, markers on LG11 in the iBS90 genome were skewed towards homozygotes, implying that iBS90 homozygotes were preferred under selection.

Revealing genetic mechanisms of schistosome-resistance in vector snails is a most important motivation for studies of snail-schistosome interactions because such knowledge may lead to innovative strategies for snail-targeted schistosomiasis control. The studies have been focused on the model snail *B. glabrata* and four genomic regions involved in resistance have been reported^23–26^. Among them, three were identified from Guadeloupe and 13-16-R1 strains by association mapping or linkage disequilibrium (LD) mapping techniques^24–26^. An additional mapping effort applied genetic crosses between outbred M line and BS90 snails (which are different from the homozygous iM line and iBS90 snails used in the present study) and randomly amplified polymorphic DNA (RAPD)-based bulked segregant analysis (BSA)^23^. In the present study, we utilized F2 individuals derived from our two homozygous lines, ddRADseq genotyping, and LOD-based genetic analysis to identify two major QTLs, qRS-2.1 and qRS-5.1.

Our comprehensive genome-wide marker analysis has already demonstrated that qRS-2.1 is a large genomic locus (not a single gene locus or marker) showing an overdominance effect for susceptibility (or underdominance effect for resistance), a genetic effect not often considered in *B. glabrata*. To further validate this mode of genetic effect, we investigated odds of infection based on the number of infected and not infected snails, as well as an F-test based on the density (read counts) of schistosome parasites present in the snail body in the SNP marker m2-37212557 of QTL qRS-2.1. The odds of infection for heterozygotes (AB) (susceptable snails (SS) = 45; resistance snails (RS) = 21) is significantly higher than that for either AA (suscepitble alleles) homozygotes (SS = 10; RS = 16) or for BB (resistance alleles) homozygotes (SS = 5; RS = 19) (45/21 > 10/16; *P* < 0.05; 45/21 > 5/19; *P* < 0.05) (detailed data provided in Supplementary Data 2). Furthermore, we have analyzed the density of parasites in snails with different genotypes (Supplementary Data 2) using density/abundance of parasite reads present in the snail body which was confirmed as an excellent phenotype of resistance/susceptibility for QTL mapping (see Fig. 6). Apparent overdominance was shown based on calculations showing that individuals with heterozygous combinations of alleles had significantly higher parasite read counts (i.e., more parasites in snail body) than either of the homozygous combinations of the alleles at the marker m2-37212557 (mean parasite density AA = 0.21 ± 0.05, AB = 0.35 ± 0.03, BB = 0.12 ± 0.05, F_2,113_ = 8.87, *P* < 0.001). Together, the two independent tests focused on the marker m2-37212557 as presented here suggested a mode of genetic effect of overdominance for susceptibility (or underdominance for resistance), which is in agrement with our conclusion based on genome-wide Simple Interval Mapping described in the Results section.

**Fig. 7:**
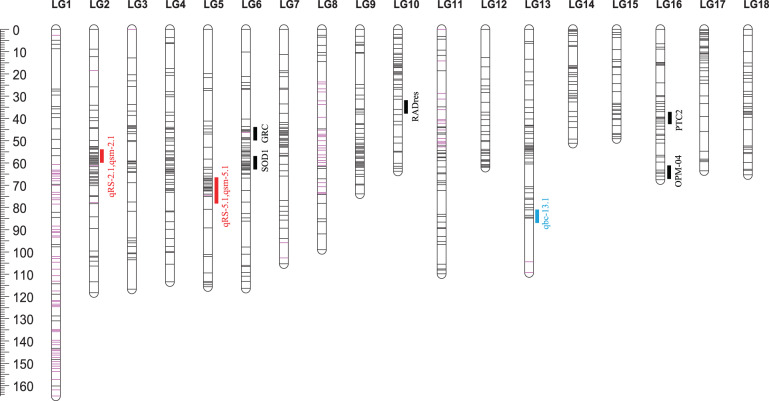
Distribution of QTLs and SNP markers on the 18 LGs. The markers derived from segregating normal and segregating distorted regions are presented as black and pink, respectively. Red solid bars indicate the QTL regions of snail resistance to parasites, and the blue solid bar show the QTL region for pigmentation identified in this study. The black solid bars indicate the QTL regions reported previously. OPM-04^23^, GRC^24^, RADres and SOD^25^, PTC2^26^. The RAPD marker OPZ-11 is not indicated because it is a repetitive sequence which could not be unambiguously mapped.

Among all 18 LGs, qRS-5.1 showed the highest LOD profile and like the other previously reported QTLs in *B. glabrata*, is associated with resistance to schistosomes (additive effect of resistance). In addition, we also noted loci with high LOD scores (but < 4) in LG4 and LG10. LG10 was found to harbor the previously identified RADres resistance locus^25^. It is intriguing that our two most prominent QTLs (qRS-2.1 and qRS-5.1) are different from, and are even on different chromosomes than, the QTLs identified by the previous studies (Fig. 7). Several plausible reasons may explain the differences. First, genetic background and allelic effects may differ among strains or cross populations (i.e., M line/BS90 crosses^23^, five-generation selected snails that originated from Guadeloupe^24^; 13-16-R1 strain^25,26^, F2 snails bred from iM line/iBS90 in current study), due to interactions between parental alleles and genetic background^47^. Second, interaction between host and parasite may be affected by genotypes of both host and parasite, and the schistosome strains used in previous studies and the present study differ^24,25^. Third, the degree of associations between a genetic marker and resistance trait in natural populations results from historical recombination which may vary among populations, whereas linkage mapping infers recombination event in populations derived from control crosses between phenotypically divergent strains^48,49^.

The two QTLs we identified are in the low-recombining centromeric and pericentromeric regions of LG2 and LG5 (Supplementary Fig. 8). The clustering of QTLs in the centromeric and pericentromeric regions has also been observed in many plants, such as root-related traits of wheat^50^, grain weight of rice and wheat^51,52^, and head blight resistance of wheat^53^. In *Drosophila*, a QTL affecting knockdown resistance to high temperature in *D. melanogaster* and a QTL for *D. sechellia* resistance to toxins were found to be strongly associated with the pericentromeric regions of the third chromosome^54,55^. In *B. glabrata*, two resistance loci, GRC/SOD (in LG6) and RADres (in LG10), were found in a low-recombining centromeric/pericentromeric region^24,25^. As noted by Tennessen et al.^25^, this region consists of a large haplotype block showing little recombination and containing several immune-relevant genes. Therefore, these haplotype blocks in the regions of low recombination may represent the combined effect of multiple contributing loci^56^. Gene ontology (GO) enrichment analysis of candidate genes within the 95% confidence intervals of QTL regions indicated that several functional candidate genes are involved in binding functions (RNA-binding, DNA binding, ATP binding, and zinc finger Ran-binding), cellular and membrane function (transmembrane and epithelial membrane). This finding is partially consistent with recent studies suggesting that transmembrane proteins play a role in resistance to infections in *B. glabrata*^24,26^. Although most of genes identified in QTLs are uncharacterized or unknown in defense, we examined 49 genes from these two QTLs and found 28 are significantly upregulated in BS90 snails at 12 h post exposure to *S. mansoni*^57^. Functional investigations (e.g., RNAi-knockdown and/or CRISPR-mediated knockout) of these genes, especially those located at the peak of LODs, are needed.

In addition to the resistance/susceptibility loci, we revealed a locus that influences snail pigmentation. In this locus, we identified a gene which is homologous to the G protein-coupled receptor NlA42. This gene has been found to play an essential role in melanization of the brown planthopper *Nilaparvata lugens*^58^, but its role in molluscan species is unknown. Identifying QTL and genes that are involved in pigmentation has not yet been reported in the Mollusca, including schistosome vector snails.

As this study utilized our newly developed genetic and genomic resources, two independent phenotype data (cercarial shedding and abundance of schistosome reads), and powerful ddRADseq-based genotyping, we believe our data are highly reliable. As resistance is controlled by multiple loci, it is highly likely that other loci may also be involved in resistance^31,32^. The effect of different loci on schistosome resistance may vary, likely depending on the snail or schistosome strains.

We are also aware of some potential limitations for genetic mapping presented in this study. First, the QTLs were mapped to large genomic regions in this study due to low map resolution resulting from the limited number of crossovers of the F2 population. Further effort will be made by applying advanced genetic resources such as recombinant inbred lines (RILs) or lines from an advanced intercross because these genetic resources will increase mapping resolution through the accumulation of additional meiotic crossover events. Second, a relatively small population size of 116 F2 snails in this study may not have provided enough power to detect QTLs with modest effect. In this study, the power of QTL detection within the 5 cM support interval was around 80% at a Type I error rate *α* = 0.01 to explain 10% of the phenotypic variance in the 116 F2 individuals, based on the calculation of the statistical power for detecting a QTL located in a marker interval^59^. Clearly, a large sample size will further improve power for QTL detection. Third, testing offspring that are bred from parental strains may also affect the outcome of QTL assignment. Since parental strains are unlikely to contain segregating alleles of large effect at every locus contributing to variation in the mapping population, some novel QTLs will remain undetected if the mapping population contains no segregating alleles at the loci. Use of population-based genome-wide association studies (GWAS) may be an option^60,61^.

In conclusion, this study selected two homozygous lines of the snail *B. glabrata* with contrasting resistance phenotype, generated two high-quality genome sequences, provided high-coverage linkage map, and identified new QTLs involved in schistosome resistance and pigmentation in snails. These genetic and genomic resources lay a foundation for further molecular studies of snail-schistosome interactions and yield new insights into mechanisms of snail resistance/susceptibility to schistosomes, which may ultimately benefit the development of innovative strategies for schistosomiasis control.

## Methods

### Ethics statement

Mice were used to maintain the schistosome life cycles and to produce miracidia for schistosome-exposure experiments. Infection is through contact with cercariae-containing water and involves minimal discomfort. Infected mice are euthanized with CO_2_ prior to showing clinical signs of disease and are dissected to recover parasitic eggs. The animal facilities and operations are maintained in compliance with the Animal Welfare Act regulations of the U.S. Department of Agriculture and guidelines for Public Health Service and Use of Laboratory Animals. The use of the mice was approved by the University of New Mexico Institutional Animal Care and Use Committee (IACUC protocol # 19-200895-MC).

### Breeding the two homozygous snail lines

The new lines, iM line and iBS90, described in the paper were bred from *B. glabrata* M line and BS90, respectively. Breeding the new selfed M line and BS-90 snail lines was started in 2001 and 2011 in our laboratory, respectively. Producing such highly inbred lines has proven difficult in many species because inbreeding depression results in a cessation of egg-laying. With careful maintenance, our homozygous snails are able to survive and reproduce. To avoid losing this unique resource, we stopped selfing the iM line at 81 generations of consecutive self-fertilization, in December 2018. Since then, this genetically stable new iM line has been well-established in our laboratory. We have continued breeding new iBS90 snails by selfing, which have now undergone 39 consecutive generations of self-fertilization. We have selected and tested the resistance/susceptible phenotype of either line from the beginning of the breeding experiment. For iM line, 10 juvenile snails (at 3–5 mm of shell diameter) were exposed to *S. mansoni* at a dose of 20 miracidia per snails in every generation at the first 20 generations and after that, the same test/selection was done in every 5 generations. The same protocol was also applied to iBS90, except more frequently. At the first 20 generations, every generation was tested and selected. From 20 generations on, the test was conducted every two generations. The two well-established genetic lines were not only used for genome sequencing, but also for production of F2 offspring for subsequent genetic mapping. Supplementary Fig. 1a provides the scheme for breeding of the two lines as well as producing F2 snails for linkage and genetic mapping.

### Generating F2 snails and testing their phenotype of schistosome-resistance

*Biomphalaria glabrata*, a hermaphroditic species, can self-fertilize, but is a preferential out-crosser. To make sure the F2 offspring was produced by crossing of the two parent snails (iM line (78-generations of consecutive selfing) and iBS90 (28-generations of consecutive selfing) and not by self-fertilization of either parent, the following procedures were used. Single iM line and iBS90 snail were placed in a plastic cup (1 L) and allowed to produce F1 progeny. As pigmentation is a dominant Mendelian trait^60^, three possible outcomes are expected in F1 snails; albino F1 snails produced by selfing of M line snails, pigmented F1 snails derived from selfing of iBSS90, and pigmented F1 derived from a cross between iM line and iBS90. Albino F1 snails were discarded and pigmented F1 snails were kept for producing F2s by selfing. To separate the two types of pigmented F1 snails, we examined F2 snails. If all F2 snails derived from one F1 by selfing were pigmented, these F2 snails were not used for genetic studies because they were from an F1 snail that resulted from self-fertilization of a parental iBS90 snail. If F2 snails were mixed with albino and pigmented ones, it indicated that their parental F1 snail was produced from a cross between iM line and iBS90 parentals. Accordingly, these F2 snails were kept for phenotype and genotype tests.

The F2 juveniles (3–5 mm shell diameter) were exposed individually to 15–20 miracidia of the PR1 strain of *Schistosoma mansoni*. Since cercariae play a decisive role in infection and transmission to the definitive hosts, we define the resistance or susceptible phenotype here as to whether the schistosome-exposed snails shed cercariae. Shedding of cercariae was conducted at 45 days post-exposure (DPE). To determine whether they were truly resistant (some snails may shed later), additional shedding was conducted at 55 DPE. The snails that did not shed cercariae at 55 DPE were considered as resistance snails whereas snails that shed cercariae at any time were recorded as susceptible ones. After screening for the cercarial shedding, each F2 snail labeled with body color (pigmented or albino) and phenotype (resistance or susceptible) was placed individually in a 1.5 ml tube with CTAB solution^62^ and then stored at −80 °C.

### Snail lines used

Only two specimens for iM line and two for iBS90 were used for whole genome sequencing; one was sequenced using Illumina technology the other was done by PacBio Sequel II. For Illumina sequencing, the single iM line or iBS90 snail was collected at 74 or 18 generations of consecutive self-fertilization, respectively. For PacBio sequencing, snails from 81 or 30 generations of self-fertilization were collected from the two lines (Supplementary Fig. 1a).

### Illumina sequencing

Genomic DNA was extracted from a single iM line or iBS90 snail (the intestine excluded) using CTAB^62^. DNA quality was assessed by agarose gel (1.0%) electrophoresis. Concentration and purity of the extracted DNA were evaluated using a Nanodrop 2000 spectrophotometer and a Qubit 3.0 Fluorometer (ThermoFisher Scientific). After selection of size (400–700 bp) using Blue Pippin, a 150 nt x 2 paired-end library was prepared (KAPA Hyper Prep Kit Illumina platforms, KAPA Biosystems, www.kapabiosystems.com) and sequenced on the Illumina NextSeq500 platform at the University New Mexico (UNM) Biology Department’s Molecular Biology Facility (http://ceti.unm.edu/core-facilities/molecular-biology.html).

### PacBio sequencing

One specimen from either iM line or iBS90 was used. DNA extraction was done using the Qiagen Genomic-tip kit (www.qiagen.com). The snail was first dissected to remove the intestine, and then flash frozen in liquid nitrogen. The snail was ground in a mortar and pestle in liquid nitrogen and the powder was suspended in a solution of 30 mM Tris pH 8, 10 mM EDTA, 1% SDS with Proteinase K (Qiagen). This was incubated at 50 °C for 30-60 min. DNA was isopropanol precipitated, washed in 80% ethanol, and then suspended in Qiagen genomic tip digestion buffer. The process was repeated as per Qiagen instructions, including the column cleanup. Library preparation was performed using the PacBio express kit 2.0 and instructions. Sequencing was performed following the PacBio Sequel II annealing/binding and cleanup instructions as found on SMRT link 7. PacBio Sequel II - SMRT Cell sequencing was conducted at Brigham Young University DNA Sequencing Center (https://biology.byu.edu/dnasc).

### Genome assembly

The overall workflow for bioinformatics and genetic analysis is provided in Supplementary Fig. 9. To generate high-quality Illumina paired-end reads, Illumina raw data were trimmed and filtered using Trimmomatic v0.36^63^, with options of slide window of 4 nt, average base quality score 20 and minimum read length 36 nt cut-off. Decontamination of Illumina reads was performed using Kraken v2.0.8 with 8GB database of reference microorganisms^64^. PacBio sequences were corrected, trimmed and assembled to contigs using assembler Canu 1.9^65^ with options (useGrid = true corOutCoverage = 100 genomeSize = 916 m). The assembled contigs were then corrected with cleaned Illumina paired-end sequence data using program Pilon 1.23^66^. The corrected contigs were further linked to scaffolds using SSPACE 3.0^67^ based on the hint of clean Illumina paired-end sequences.

To check contaminations in initial assembly, modified workflow from Blobtools^68^ were used. Scaffold sequences were split into 10 Kbp fragments using in house bash/perl scripts, which were compared against NCBI non-redundant nucleotide database using the BLASTn program in NCBI-BLAST + package 2.11.0^69^ with options: -task megablast -outfmt ‘6 std qlen slen staxids stitle’ -num_threads 79 -max_target_seqs 1 -max_hsps 1 -evalue 1e-5 -perc_identity 60. The BLAST output file was loaded into Blobtools to classify hits taxonomically at the phylum level for each scaffold. Scaffolds were marked as contaminated if phylum was assigned to bacterial genomes and found no hits to known Mollusca nucleotide sequences. Final scaffolds without strong enough signatures of contaminations were sorted based on length and renamed. Evaluation of core conservative genes in genome assembly were performed using Benchmarking Universal Single-Copy Orthologs BUSCO 4.1.4 with metazoan core gene dataset metazoa_odo10^30^.

### Genome annotation

Genome annotation for the two strains was conducted independently in three stages: (1) repeated mask to identify repeat elements; (2) gene model prediction in genomic regions outside of repeated regions; (3) functional annotation or assigning functions to predicted gene models. Repeats in genome were initially identified and classified using RepeatModeler 2.0.1^70^. The predicted repeat models were then checked to keep retrotransposon-related functional domains and to exclude any other functional domains predicted by InterProScan 5.45^71^. Clean repeat models were used to mask the genome using RepeatMasker 4.0^72^. Based on the repeats identified, the scaffolds were soft masked (repeats in lower-case letters) for gene prediction.

Gene models were predicted using ﻿EVidence Modeler 06/25/2012^73^, by integrating weighted evidence from ab initio predictions, RNA sequencing (RNA-Seq) alignments, and homology-based searches.

The ab initio prediction results were collected from the output of BUSCO, which identifies core single copy orthologs using customized HMM models built from tBLSATn^69^ and AUGUSTUS predictions^74^. Annotation evidence was a collection of sequences merged from predicted transcripts of the *B. glabrata* BB02 genome, de novo assembled transcriptomes of tissue-specific RNA-Seq data^27^, and recent schistosome infection studies (RNAseq) on *B. glabrata* M line and BS90 strains^75,76^. Redundant transcripts were removed using CD-HIT-EST^77^. Protein sets were derived from the merged transcripts using EvidentialGene^78^. Sequences showing similarities (E value <10^−5^ and coverage >50%) to known repeat elements (RepeatPeps.lib in RepeatMasker libraries) were filtered out by BLASTp^69^.

Non-redundant transcript sequences generated above served as evidence in the Program to Assemble Spliced Alignments (PASA) pipeline^79^, which infers gene models consistent with the spliced alignments of expressed transcript sequences.

The RNA-Seq data from 12 tissues^27^ and infection studies^75,76^ were also used to aid the annotation. This involved sequence cleaned up using Trimmomatic v0.36 as described above in genome assembly, then alignment to the assembled genome using HISAT2^80^. The alignment files and evidence transcripts were used as input for the BRAKER 2.1.1 annotation pipeline^81^, in which GeneMark-ET^82^ utilizes RNA-Seq reads to genome alignments and transcripts to genome alignments to generate annotation hints to train AUGUSTUS, in two rounds: first separately, then merged in a second round as described in the BRAKER2 pipeline.

For functional annotation, the coding sequences and protein sequences based on the predicted gene models were searched against reference databases: (1) BLASTp to the UniProt database^83^ with minimum identity of 30%, max E value 10^−5^, and minimum aligned length 10 aa; (2) BLASTp to the NCBI non-redundant protein database (NR) with the same cut off above^84^; (3) BLASTn to the NCBI non-redundant nucleotide database (NT)^83^ with minimum identity at least 60%, maximum E value 10^−5^; (4) conserved functional domain by InterProScan 5.45^71^, and *Biomphalaria* specific IgSF domains predicted by *B. glabrata* specific HMM model with minimum length of 40 aa, and max E value 0.001^76^.

### Genomic structural variation analysis

To identify synteny and structural variations (SV) between iM line and iBS90 genomes, the draft genome of iBS90 was aligned as query using the iM line genome as reference, using minimap2 version 2.17-r941^85^ with options (-ax asm5 --eqx). The scaffolds fell into 18 linkage groups of iM line and their corresponding syntenic scaffolds in iBS90 were selected for the following steps. The output alignments in SAM format were used to identify structural variations using SyRI^86^ with options (-k -F S). Annotated genes overlapping with predicted SVs were filtered using bedtools v2.29.2^87^. Synteny and SVs results data were organized and visualized using R packages openxlsx_4.1.5^88^, tidyverse_1.3.0^89^, and ggplot2_3.3.2^90^.

### Double digest restriction-site associated DNA sequencing (ddRADseq)

Genomic DNA was extracted individually using CTAB and re-purified using the mollusk DNA kit (www.zymonresearch.com). Quantity and quality of the DNA were checked using Nanodrop and Qubit, respectively. Genomic DNA was double-digested with the restriction enzymes, *Ecor* I and *Msp* I to prepare the ddRADseq libraries. The unique barcode of 8 nucleotide base pairs with an Illumina adaptor was assigned to each individual accession for tracing the samples. The adaptor-ligated DNA amplicons were pooled and DNA fragments of 400–600 bp length were selected for library constructions. The ddRADseq libraries were sequenced on the MiSeq Illumina platform using 2 × 150 bp paired-end (PE) mode at Admera Health (www.admerahealth.com). A total of 122 F2 snails and 4 parent snails (2 iM line and 2 iBS90) were analyzed by the ddRADseq.

### ddRADseq data processing and SNP genotyping

Sequence reads were de-multiplexed by MiSeq Reporter software and assigned to sequenced individuals. Sequence quality was checked using FastQC v. 0.11.9^91^. Sequence trimming was performed using Trimmomatic v. 0.36^63^ to filter poor quality reads, and to remove adapters, barcode primer as well as some universal primers. Reads were also trimmed if 10 bases within a sliding window fell below a Phred score of Q20, or if index barcode, enzyme-specific adapter or Illumina adapter sequences were present, indicating read-through beyond sequenced insert fragments. After reads cleaning, the high-quality reads with a minimum length of 36 bp of 126 samples were individually aligned to the two reference-genome iM line and BS90, respectively, by using the Burrows-Wheeler Alignment tool (BWA)^92^ with default parameters. In addition, the 4 parents pooled reads were also aligned to the two reference genomes. The alignments were converted to Sequence Alignment Map (BAM) format and duplicated reads were removed with Samtools 1.2^93^.

To determine polymorphisms of SNP markers between resistant and susceptible parents, genomic regions of read mapping from four parents with coverage between 40 and 4000 reads were extracted using the ‘Create Mapping Graph’, ‘Identify Graph Threshold Areas’, and ‘Calculus Track’ tools in CLC Genomics Workbench 12.0 (QIAGEN). SNP calling was performed on the coverage-restricted regions for all four parent samples, using the ‘Fixed ploidy variant detection’ tool in the CLC. The VCF file obtained in CLC from read mapping of the parent samples was used as the variant track file.

The ‘Identify Known Mutations from mappings’ tool within the CLC was used to genotype the 122 F2 samples individually at the SNP loci in the VCF file. The following parameters were used to filter variants: a minimum coverage of 5, minimum frequency of 20%, Ignore non-specific matches = Read, Ignore broken pairs = No, Include partially covering reads = No. The SNP allele specific to the resistant or susceptible parent, in which parents must be homozygous and have different genotypes, and those segregating in F2 population were considered genetically informative for genetic linkage mapping construction. SNP allele frequency ranging from 20% to 80% were classified as heterozygous while those <20% and >80% were classified as homozygous alleles derived from a resistant parent and a susceptible parent, respectively^94^. The genotype data were converted from a nucleotide-based format to a parent-based format as ABH in IciMapping. The threshold of maximum genotype missing rate was set to 25%. SNPs with missing genotypes in either parent were also excluded from further analysis. The markers were named based on the scaffold # and the physical position of the SNP.

### Linkage map construction and QTL analysis

To construct the genetic linkage map, PLINK^95^ and Haploview^96^ were used to analyze linkage disequilibrium (LD) and select tag SNPs that represent the genome-wide markers. Pearson’s Chi-squared test was performed to examine the goodness of fit to the expected 1:1 segregation ratio (*P* < 0.01) of alleles and 1:2:1 ratio of genotypes segregating in the F2 population. The MAP functionality in QTL IciMapping version 4.2.53^97^ was used to construct the linkage map: SNP markers were grouped using a logarithm of the odds (LOD) threshold of 8.0 and a recombination frequency of 0.14. Linkage groups (LGs) with 10 or less markers were deemed to be unlinked and dropped from further construction. Marker ordering within group was based on their physical position. Recombination frequency was determined with window size of 5. Recombination frequencies between markers were converted into centiMorgans using the Kosambi mapping function^98^. The best marker order with the shortest linkage map distance was selected, and a linkage map was constructed with the information regarding marker segregation based on chi-square goodness of fit. The software MapChart^99^ was used to display the marker names and genetic distance on the linkage map. More unassigned scaffolds were added to linkage groups if the visualization of synteny between iM line and iBS90 scaffolds suggested the presence of a continuity bridge.

The R/qtl and R/ASMap packages in R Statistical Computing Environment were used to calculate recombination fractions and LOD scores for all marker pairs. The function pull.rf() in R/qtl package was used to pull out the recombination fractions and LOD scores as matrices. A plot of the LOD scores against the estimated recombination fractions for all marker pairs was made to inspect any problem markers that had higher recombination fraction values and higher LOD scores. The function heatMap in R/ASMap package was used to create a heat map for the estimated recombinant fractions and the LOD scores.

QTL detections were performed using BIP functionality in QTL IciMapping. The functions for conventional simple interval mapping (SIM) and inclusive composite interval mapping were selected to detect additive QTL and dominance QTL. The mapping parameter step was set at 1.0 cM, and the LOD threshold for QTL declarations was determined with a 1000 permutation test at a 95% confidence interval, which controls the number of false positives, known as Type I error^36^. A LOD support interval of 1.0-LOD from the peak LOD was used to define QTL interval with 95% coverage^100^. QTLs were designated according to standard nomenclature based on the trait name and chromosome location. For example, qRS-5.1 is the first QTL for snail resistance to *S. mansoni* on chromosome 5. A QTL was defined as a major QTL if percentage of variation explained (PVE) was >10% or a minor QTL if <10%.

### Marker validation

To confirm genetic effects of the major QTL qRS-5.1, 12 out of the top 20 SNP markers (based on −log10 *p*-value) located within the QTL regions were randomly selected for validation of genotypes determined by ddRADseq assay. Technical validation was performed by Sanger sequencing of 46 resistant (R) F2 snails (27 pigmentation (B) and 19 albino (W)), and 48 susceptible (S) F2 snails (29 pigmentation and 19 albino) that were phenotyped and used for ddRADseq, whereas biological validation was conducted by Sanger sequencing of an additional set of 48 randomly selected F2 snails (12RB, 12RW, 12SB, and 12SW) that were phenotyped and not used for ddRADseq. The RAPD marker OPM-04 (Genbank accession number AF078109) was also included as a comparison for allele effects on parasite infections. Primers were designed based on the flanking regions of the SNPs using Primer 3 software. List of the forward and reverse PCR primers is shown in Supplementary Table 4.

### Reporting summary

Further information on research design is available in the Nature Research Reporting Summary linked to this article.

## Supplementary information

Supplementary Information

Description of Additional Supplementary Data

Supplementary Data 1

Supplementary Data 2

Supplementary Data 3

Supplementary Data 4

Supplementary Data 5

Supplementary Data 6

Reporting Summary

## Data Availability

The raw sequences for genome assembly and ddRADseq, as well as assembled annotated genome sequences, were all submitted to NCBI project with accession number PRJNA769727.
