# Supplementary material for: Compatibility between snails and schistosomes: insights from new genetic resources, comparative genomics, and genetic mapping
Source: Commun Biol. 2022 Sep 9;5:940. doi: 10.1038/s42003-022-03844-5 (PMC9463173; doi:10.1038/s42003-022-03844-5)
Supplement: Supplementary file 1 — Supplementary Information [file 42003_2022_3844_MOESM1_ESM.pdf]

Compatibility between snails and schistosomes: insights from new genetic resources, comparative genomics, and genetic mapping

Lijing Bu[1a], Daibin Zhong[2a], Lijun Lu[1], Eric S. Loker[1], Guiyun Yan[2], Si-Ming Zhang[1*]

[1] Center for Evolutionary and Theoretical Immunology, Department of Biology, University of New Mexico, Albuquerque, NM 87131, USA

[2] Program in Public Health, College of Health Sciences, University of California, Irvine, CA, 92697, USA

[a] These authors contributed equally to this work.

[*] Correspondence: zhangsm@unm.edu

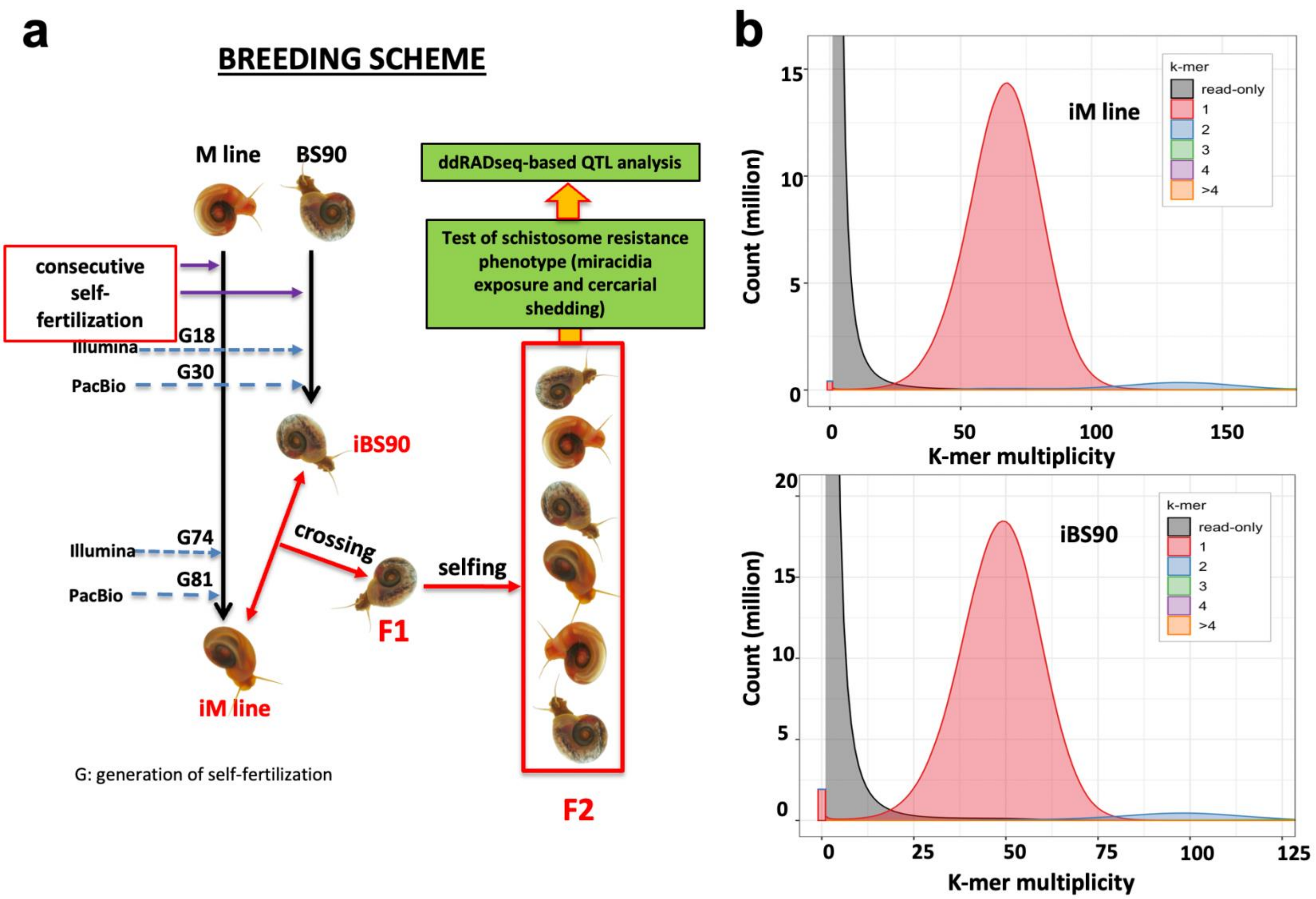

Supplementary Fig. 1. Breeding scheme of iM line and iBS90 snails (a) and K-mer test of homozygosity of the two snail lines (b). All snail images were produced by Dr. Si-Ming Zhang, the corresponding author of the paper. A single large 1x peak in iM line and iBS90 suggest homozygous genomes for the two lines.

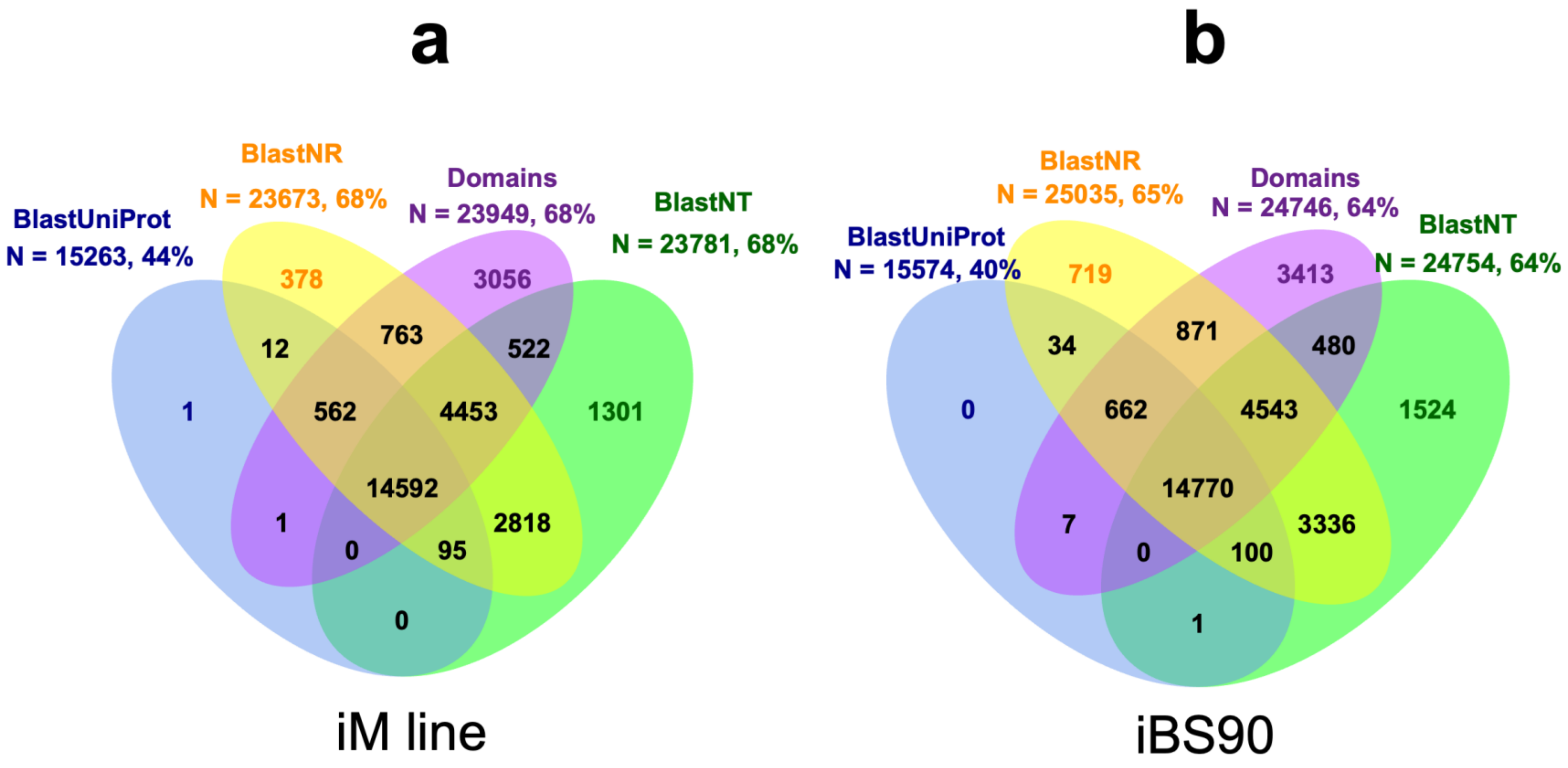

Supplementary Fig. 2. Functional annotation for predicted gene models in iM line (a) and iBS90 (b). Four sources of databases used for functional annotation: Uniports database, NCBI non-redundant nucleotide (NT) database, NCBI non-redundant protein (NR) database, and InterProScan integrated conserved domain databases. Percentages were calculated by comparing to a total of 35,015 predicted protein coding gene models in (a) iM line genome, and 38,516 in (b) iBS90 genome.

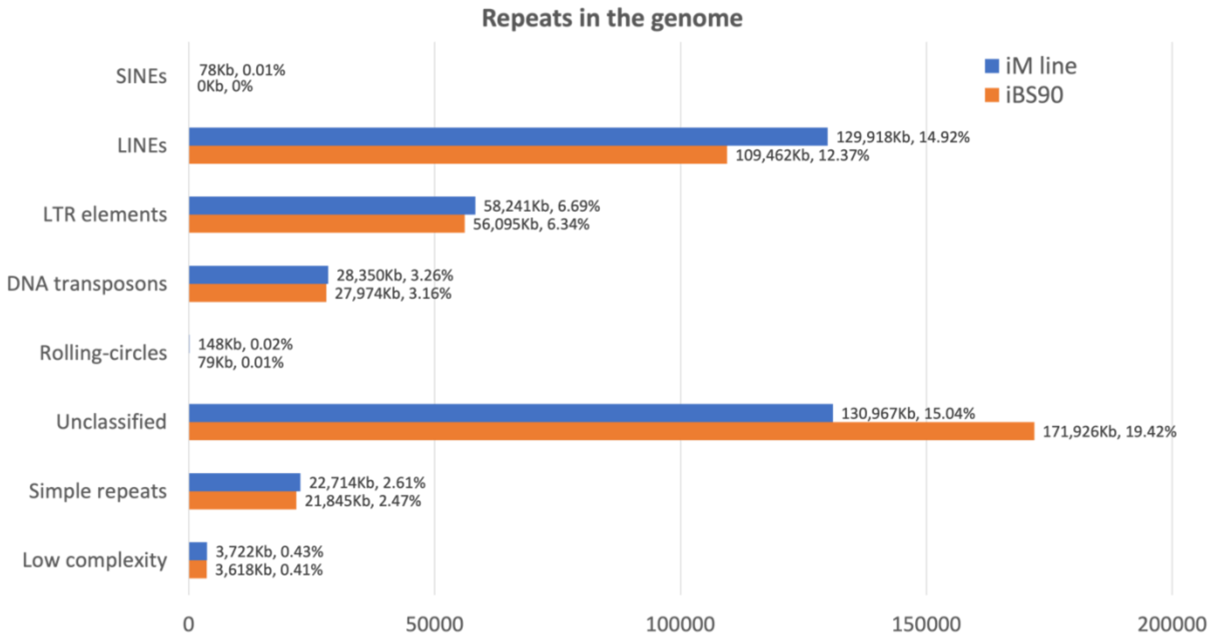

Supplementary Fig. 3. Repeats in iM line and iBS90 genomes. The total size (Kb) and its percentage in corresponding genomes listed on right of the bars. Detailed repeats families are provided in Supplementary Table 1.

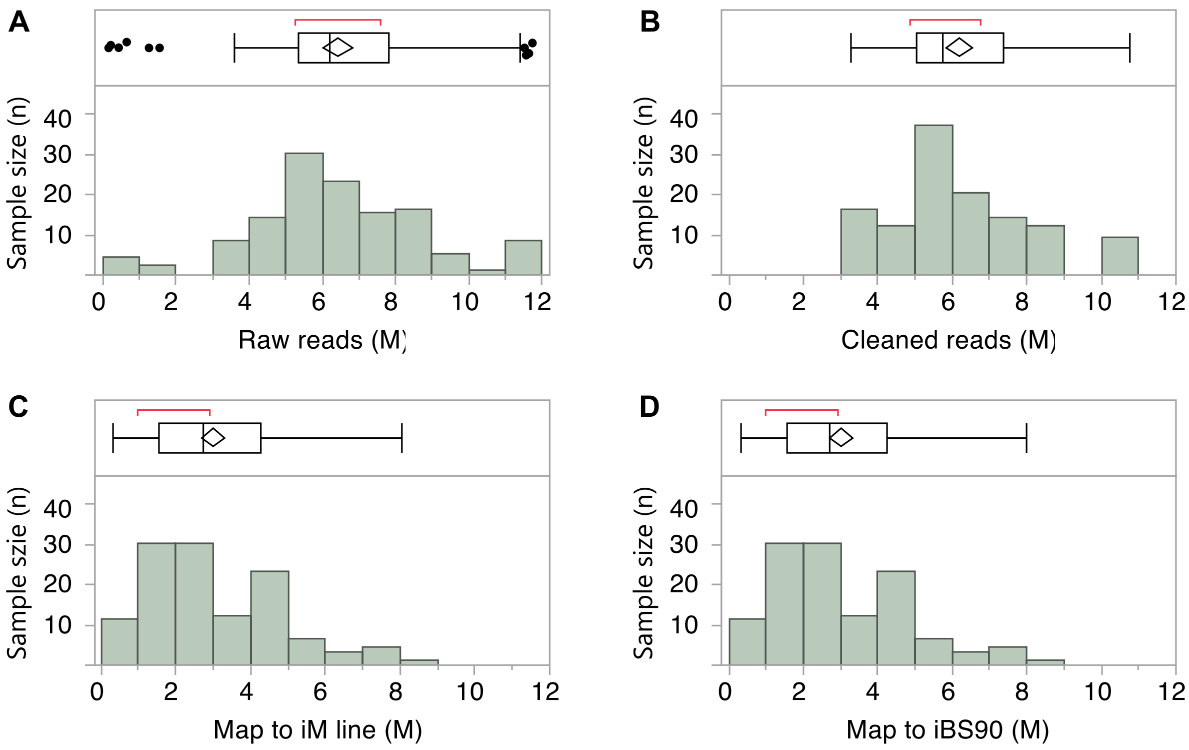

Supplementary Fig. 4. Distributions of paired-end reads in millions (M) among the samples. (**A**) raw read counts (n=126). (**B**) high quality cleaned read counts (n=120). (**C**) counts of cleaned reads mapped to iM line genome (n=120). (**D**) counts of cleaned reads mapped to iBS90 genome (n = 120). In the upper box, 25th, 50th and 75th quartiles are displayed; the mean and the 95% confidence interval are represented by a diamond. The red brackets represent the shortest half (most dense 50% of observations).

a b

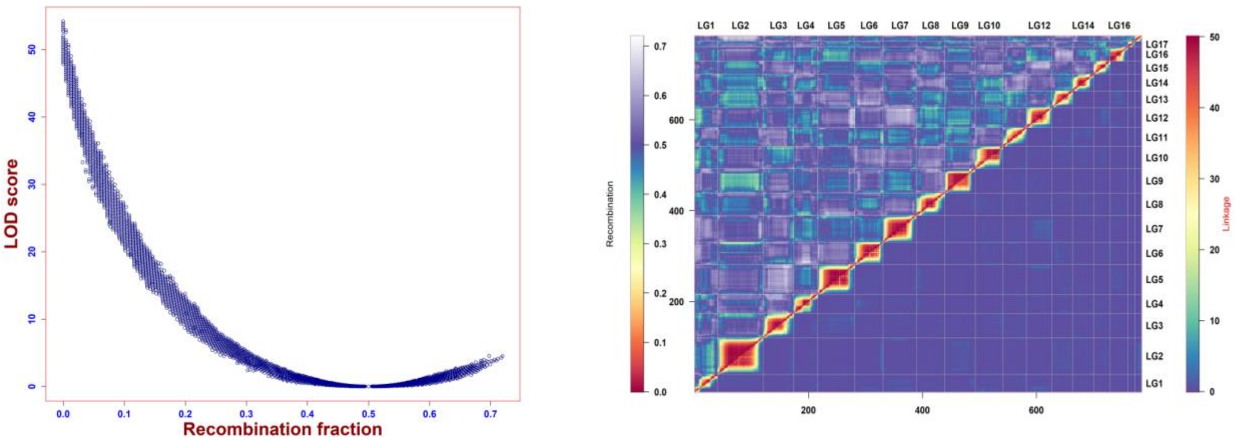

Supplementary Fig. 5. Pairwise recombination fraction (RF) and logarithm of odds (LOD) scores. (a) a plot of the LOD scores against the estimated recombination fraction for all marker pairs shows some markers have RF more than 0.5 (but the LODs are low). (**b**) a heatmap of estimated recombination fractions (upper-left triangle) and LOD scores (lower-right triangle) for all pairs of markers. Red indicates markers that are linked (large LOD score or small recombination fraction), and blue indicates markers are not linked (small LOD score or large recombination fraction).

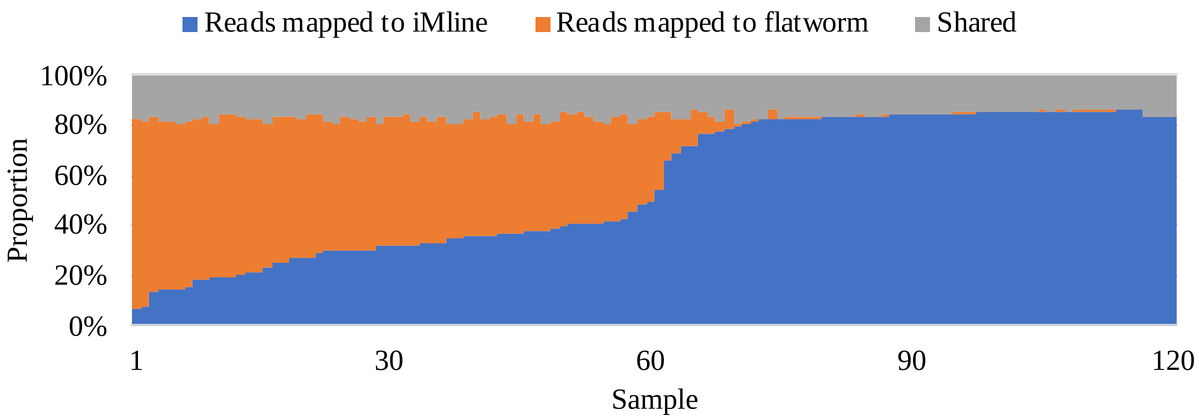

Supplementary Fig. 6. Pattern and proportion of ddRADseq reads mapped to the genomes of iM line *B. glabrata* and flatworm *S. mansoni* in individual F2 snails. Approximately 20% of reads were mapped to both snail and schistosome genome (shared). Samples #1-116 were F2 snails, samples #117-120 were 4 parents (2 iM line snails and 2 iBS90 snails).

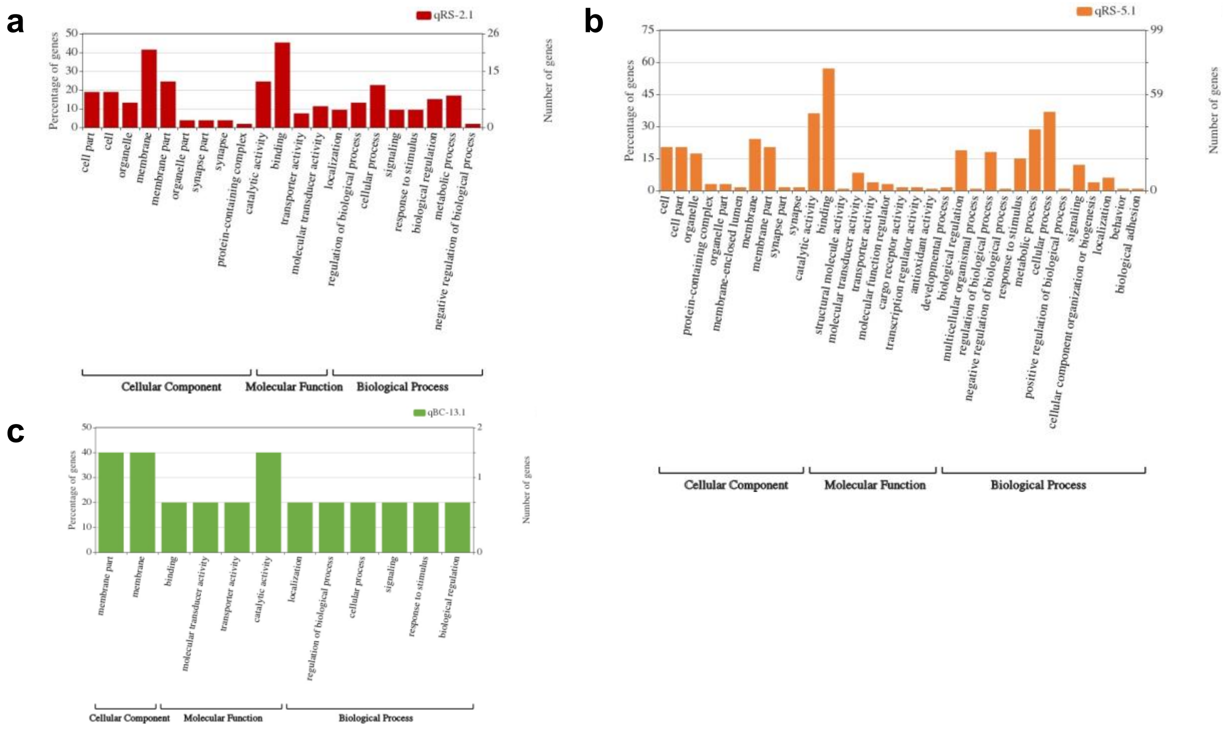

Supplementary Fig. 7. Gene ontology (GO) enrichment analysis of candidate genes within the 95% confidence intervals of QTL regions. (**a**) schistosome-resistance QTL on linkage group 2 (LG2); (**b**) schistosome-resistance QTL on linkage group 5 (LG5); (**c**) QTL of pigmentation on linkage group 13 (LG13).

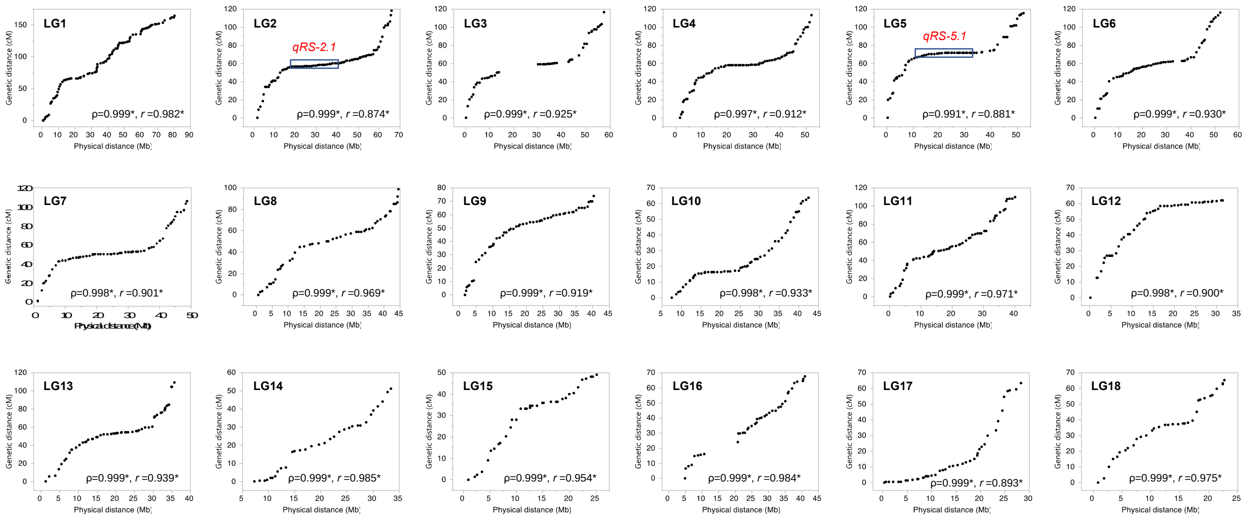

Supplementary Fig. 8. Correlation between genetic distance and physical distance for the 18 LGs/chromosomes of *B. glabrata*. Genetic position of the 996 SNP loci was plotted against the corresponding physical position. Blue box indicated QTL regions with low recombination.

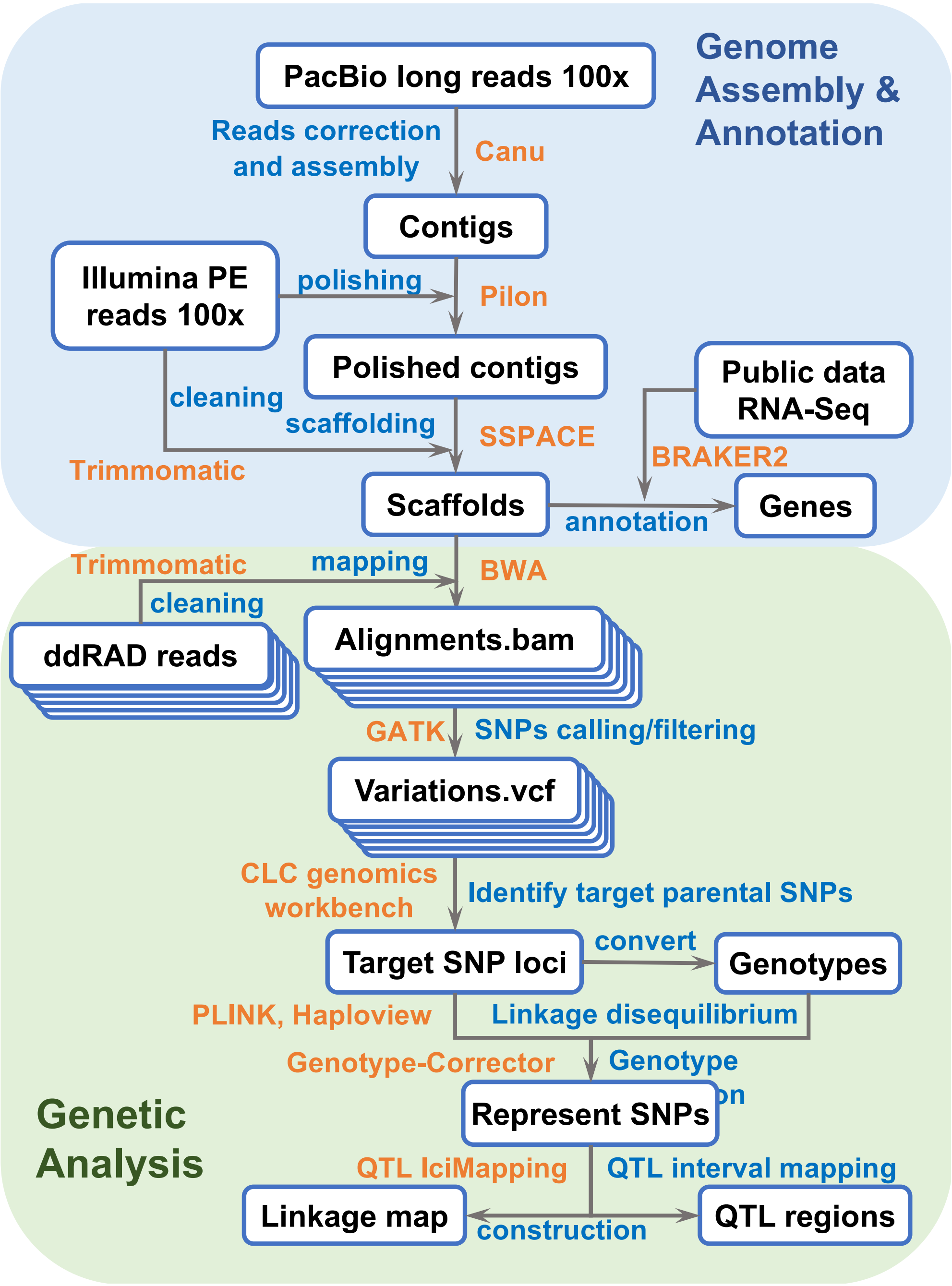

Supplementary Fig. 9. A workflow of genome sequencing and genetic analysis.

Supplementary Table 1. Summary of structural variations (SVs) between iM line and iBS90 genomes

Structural variations	Count	Length_ref	Length_qry	Length_ref%	Length_qry%
Syntenic regions	17,191	559,879,348	539,680,447	64.28%	60.97%
Inversions	188	8,618,139	8,942,609	0.99%	1.01%
Translocations	9,147	38,531,061	38,501,179	4.42%	4.35%
Duplications (reference)	878	6,183,720	-	0.71%	-
Duplications (query)	11,468	-	31,431,550	-	3.55%
Not aligned (reference)	24,911	234,318,664	-	26.90%	-
Not aligned (query)	34,266	-	205,000,387	-	23.16%
Total				97.31%	93.05%

Affected Sequences	Count	Length_ref	Length_qry	Length_ref%	Length_qry%
Insertions	794,563	-	6,081,815	-	0.69%
Deletions	526,466	6,513,551	-	0.75%	-
Copygains	654	-	1,888,992	-	0.21%
Copylosses	877	2,187,621	-	0.25%	-
Highly diverged	72,571	235,112,385	215,957,637	26.99%	-
Tandem repeats	207	395,491	386,982	-	0.04%
Total	1,395,338	244,209,048	224,315,426	27.99%	0.94%

Structure variations (SVs) were predicted by SyRI software, with all linkage group assigned scaffolds in iM line as reference (ref) and iBS90 as query (qry). The ref% were calculated based on iM line assembled size of 870,959,050 bp, and qry% based on iBS90 assembled size of 885,111,083 bp.

Supplementary Table 2. Repeat elements in iM line and iBS90 genomes

	iM line (total length of repeats: 374,059,649 bp, 42.95 %)			iBS90 (total length of repeats: 391,000,445 bp, 44.17 %)		
Repeats	number of elements [a]	occupied length (bp)	sequence %	number of elements [a]	occupied length (bp)	sequence %
Retroelements	625,829	188,158,502	21.60%	528,369	165,557,746	18.70%
SINEs:	-	-	0.00%	-	-	0.00%
Penelope	209	78,188	0.01%	-	-	0.00%
LINEs:	513,534	129,917,982	14.92%	398,678	109,462,399	12.37%
CRE/SLACS	-	-	0.00%	-	-	0.00%
L2/CR1/Rex	165,061	38,567,854	4.43%	91,120	27,474,333	3.10%
R1/LOA/Jockey	4,566	5,063,167	0.58%	19,466	5,002,175	0.57%
R2/R4/NeSL	6,890	2,767,563	0.32%	3,801	881,767	0.10%
RTE/Bov-B	303,028	73,222,080	8.41%	185,690	53,144,735	6.00%
L1/CIN4	1,323	672,542	0.08%	-	-	0.00%
LTR elements:	112,295	58,240,520	6.69%	129,691	56,095,347	6.34%
BEL/Pao	-	-	0.00%	-	-	0.00%
Ty1/Copia	321	361,728	0.04%	14,357	2,626,849	0.30%
Gypsy/DIRS1	33,704	29,985,104	3.44%	82,716	41,719,086	4.71%
Retroviral	-	-	0.00%	-	-	0.00%
DNA transposons	137,883	28,350,202	3.26%	111,919	27,974,457	3.16%
hobo-Activator	124,111	25,599,221	2.94%	101,118	24,954,541	2.82%
Tc1-IS630-Pogo	2,380	482,752	0.06%	1,204	340,511	0.04%
En-Spm	-	-	0.00%	-	-	0.00%
MuDR-IS905	-	-	0.00%	-	-	0.00%
PiggyBac	1,168	492,174	0.06%	1,077	474,240	0.05%
Tourist/ Harbinger	-	-	0.00%	-	-	0.00%
Other (Mirage, P-element, ransib)	270	105,939	0.01%	-	-	0.00%
Rolling-circles	257	147,860	0.02%	52	79,365	0.01%
Unclassified:	725,333	130,967,078	15.04%	878,614	171,925,546	19.42%
Total interspersed repeats		347,475,782	39.90%		365,457,749	41.28%
Small RNA:	-	-	0.00%	-	-	0.00%
Satellites:	-	-	0.00%	-	-	0.00%
Simple repeats:	341,491	22,714,214	2.61%	339,319	21,845,229	2.47%
Low complexity:	45,846	3,721,793	0.43%	44,925	3,618,102	0.41%

[a] most repeats fragmented by insertions or deletions have been counted as one element.
Repeats were identified using RepeatMasker 4.0, with customized repeat libraries, which was generated using RepeatModeler 2.0.1 followed by filtering out protein like sequences using BLAST and InterProScan domain prediction (details in Methods).

Supplementary Table 3. Genotype validation of alleles for schistosome resistance and body pigmentation in the F2 snails.

	Technical validation			Biological validation		
SNP marker	**n**	**OR [95% CI]**	**OR_P**	**n**	**OR [95% CI]**	**OR_P**
m8-1096014[a]	81	3.29 [1.72,6.31]	2.58E-04	47	3.20 [1.37,7.49]	6.53E-03
m8-8345551 [a]	92	3.35 [1.83,6.14]	6.86E-05	46	3.22 [1.37,7.57]	6.47E-03
m8-9280536 [a]	64	3.84 [1.83,8.07]	2.89E-04	47	3.44 [1.47,8.05]	3.73E-03
m8-13629664 [a]	94	3.77 [2.06,6.90]	1.16E-05	46	3.53 [1.49,8.33]	3.48E-03
m8-13659857 [a]	93	3.84 [2.09,7.05]	1.02E-05	46	3.64 [1.53,8.67]	2.98E-03
m12-23747641 [a]	87	3.82 [2.04,7.17]	2.12E-05	47	3.15 [1.35,7.35]	6.91E-03
m15-17387971 [a]	94	2.27 [1.27,4.08]	5.51E-03	47	2.39 [1.04,5.47]	3.81E-02
m2-38804054 [d]	88	4.13 [1.17,3.95]	5.50E-03	46	4.35 [1.36,7.50]	2.77E-02
m2-39800184 [d]	87	3.33 [1.78,6.24]	3.52E-04	46	3.53 [1.49,8.33]	1.04E-02
m49-1163967 [a]	92	5.27 [2.73,10.15]	2.78E-07	45	4.67 [1.89,11.52]	5.98E-04
m49-1796291 [a]	90	8.08 [3.94,16.54]	1.28E-09	45	6.69 [2.65,16.87]	2.84E-05
m49-2595029 [a]	91	11.12 [4.86,25.42]	2.20E-10	46	8.10 [3.07,21.36]	8.00E-06

Technical validation was performed by Sanger sequencing of a total of 94 F2 snails (27RB,19RW,29SB, and 19SW; R: resistance, S: susceptible, B: pigmentation (black), W: albino (white)) randomly selected from those used for ddRAD-seq. Biological validation was conducted by Sanger sequencing of an additional set of 48 randomly selected F2 snails (12RB,12RW,12SB, and 12SW) that were phenotyped (shedding cercariae) and not used for ddRAD-seq. n: number of snails genotyped; OR: odds ratio; CI: confidence interval; OR_P: p-value for odds ratio; a: odds ratio was calculated based on additive effect; d: odds ratio was calculated based on dominance effect. The OR values were presented based on the best model of fit (additive or dominant effect). The markers for resistance (m8 and m12) with additive effect indicated that the susceptible parent allele increased infections, whereas resistance parent allele decrease infections. The two markers (m2-38804054 and m2-39800184) with dominant effect indicated that heterozygous alleles increased infections. The markers (m49) for pigmentation with additive effect indicated that the pigmented parent allele increased pigmentation. The validation makers that do not appear in supplementary data 3 were manually added to Fig.3 based on their physical positions.

Supplementary Table 4. PCR-primers used for validation

Marker	LG	Gene name	Forward primer	Reverse primer	Length (bp)
m8-1096014	5	neuroligin-1	ATCTGAAACCCCGAGGTCAT	CGATCCGATCCAGATCCTAC	274
m8-8345551	5	receptor-type tyrosine-protein phosphatase	GACCCCTGACCTGGATGAC	TCATTCTCCGGCTACGTTCT	165
m8-9280536	5	uncharacterized protein	ATCTGGGCTGTGCCTTACAG	GCCGTTTAGGTACAGCGAAG	368
m8-13629664	5	ferric-chelate reductase 1	ACCTTCGGACACCATTCATC	GTCATTGTCGTCCACTGCTG	199
m8-13659857	5	DNA excision repair protein	GCAAGAATTCCCAAGGAACC	CAATGGTGTGGCAGTCATCT	176
m12-23747641	5	R-spondin-2 isoform X1	GAAGGGAGAGCATCGTTCAA	CTACCCGGTGTGTCCAAGAG	876
m15-17387971	16	OPM-04 (AF078109)	GGCGGCTTATTGTTATGCAC	GGCAATAGTTCCTCGATGCT	1066
m2-38804054	2	intergenic	GGGGGAGGTGGACTATTGTT	CATGAGGCTTAGCACGTTCA	165
m2-39800184	2	intergenic	ACTTTTCCGGACGTCCTTGT	GCACAGTAGACACCGCAGAA	169
m49-1163967	13	intergenic	TCAGCTGTAGTGGCCATTCTC	CGCGACGACTTGACCTAGTTA	156
m49-1796291	13	hemocyanin-like	TGCACAGGAAGCAGAGCTTA	ATCACACAGCCATCATCAGC	190
m49-2595029	13	intergenic	GTTGTGGGGTCCGATCTTT	CTGTAAGAGCCGGTGCTTGT	151

LG, linkage group

Supplementary Table 5. Non-synonymous mutations of key genes located within the 95% confidence intervals of QTL regions

QTL	Gene ID	Coding region change	Amino acid change	Gene description[a]	GenBank acc.
qRS-2.1	iM03928	632C>A	Thr211Asn	Vacuolar protein 8-like	XP_025099013
	iM03993	418C>T	Arg140Cys	NR2	ACA13599
	iM03995	4949A>T	Tyr1650Phe	NACHT domain- and WD repeat-containing protein 1-like isoform X1	XP_025105873
	iM04064	4580T>A, 4542T>G, 4538A>C, 4510T>A, 4501T>G	Phe1527Tyr, His1514Gln, Tyr1513Ser, Phe1504Ile, Ser1501Ala	Protein crumbs homolog 1-like	XP_013093109
	iM04067	683A>G	Tyr228Cys	Sushi, von Willebrand factor type A, EGF and pentraxin domain-containing protein 1-like isoform X2	XP_013084206
	iM04093	482T>C	Val161Ala	Epithelial membrane protein 1-like	XP_013093050
qRS-5.1	iM12488	3431A>G, 3445A>C, 3461A>T	His1144Arg, Lys1149Gln, His1154Leu	NACHT and WD repeat domain-containing protein 2-like isoform X1	XP_013076357
	iM12716	146G>C	Gly49Ala	Pancreatic triacylglycerol lipase-like isoform X1	XP_013079951
	iM12779	137A>C	Lys46Thr	Platelet endothelial aggregation receptor 1-like isoform X2	XP_013081510
	iM12785	2368A>T	Ile790Phe	Receptor-type tyrosine-protein phosphatase alpha-like	XP_013096147
	iM12788	663A>C, 704A>T, 706C>G	Lys221Asn, Tyr235Phe, Leu236Val	Cell death abnormality protein 1-like	XP_013083824
	iM17103	1313G>A	Gly438Asp	Prostaglandin E2 receptor EP4 subtype-like	XP_033736976
	iM17265	230G>A	Cys77Tyr	Serine/threonine-protein kinase MARK1-like isoform X6	XP_013071435
	iM17331	8242G>A	Gly2748Ser	Protein jagged-1b-like	XP_013079318
qBC-13.1	iM32835	715C>A	Gln239Lys	Neuropeptides capa receptor-like	XP_013077196

[a] gene description was retrieved from GenBank based on BLAST sequence similarity search.

Supplementary Table 6. Number of SNPs in the major genes located at top LOD profiles of QTL regions

QTL	Gene ID	Up-stream (5 kb)	Down-stream (2 kb)	Intron	Non-synonymous coding	Synonymous coding	Total	Gene description[a]	GenBank acc.
qRS-2.1	iM03764			8			8	Neuronal acetylcholine receptor subunit alpha-10-like	XP_013079750
	iM03799			8			8	Neuronal acetylcholine receptor subunit beta-3	XP_005094753
	iM03815			1			1	Sushi, von Willebrand factor type A, EGF and pentraxin domain-containing protein 1-like isoform X2	XP_013070411
	iM03819	4					4	Hypothetical protein EGW08_015899, partial	RUS76337
	iM03820	1					1	Phosphoribosyl pyrophosphate synthase-associated protein 1-like	XP_013094897
	iM03821	3					3	Disks large homolog 5-like isoform X1	XP_013072643
qRS-5.1	iM12892			2			2	Glutamate receptor ionotropic, NMDA 3A-like isoform X3	XP_025110159
	iM13007			28		4	32	Orexin receptor type 2-like	XP_013076003
	iM13008	2					2	Orexin receptor type 2-like	XP_013076003
	iM13016			20		1	21	Putative ferric-chelate reductase homolog	XP_013076467
	iM13020			2			2	Peroxidasin homolog	XP_013091598
qBC-13.1	iM32827			1			1	ATP-dependent DNA/RNA helicase DHX36	XP_012944942
	iM32835		5		1		6	Neuropeptides capa receptor-like	XP_013077196
	iM32843			20			20	ADAM family mig-17-like	XP_013080270
	iM32846	5					5	A disintegrin and metalloproteinase with thrombospondin motifs 7-like	XP_013063629

[a], gene description was retrieved from GenBank based on BLAST sequence similarity search.

Supplementary Table 7. A comparison of linkage groups between current work and a reported study

LG	LG1	LG2	LG3	LG4	LG5	LG6	LG7	LG8	LG9	LG10	LG11	LG12	LG13	LG14	LG15	LG16	LG17	LG18
LGI	1457	45	21	27	40	26	119	45	22	13	26	23	34	12	19	19	17	16
LGXX	11	1	0	0	1	0	1	0	1	0	0	0	0	0	1	0	0	1
LGII	21	846	18	117	22	16	14	34	17	35	30	19	12	16	15	21	11	16
LGIII	29	9	795	27	17	11	34	8	15	16	13	52	9	12	10	7	12	16
LGIV	17	15	21	1114	14	15	45	13	12	14	57	11	11	10	7	11	14	10
LGXXII	0	0	0	13	0	0	0	0	0	0	0	0	0	0	0	0	0	0
LGV	11	17	11	11	610	12	52	27	13	6	11	12	10	6	9	10	7	5
LGIX	18	14	4	10	11	357	15	3	7	7	9	11	10	7	6	5	4	4
LGVI	12	11	12	9	9	30	1009	10	10	35	56	10	11	10	7	8	27	6
LGVIII	19	4	9	4	13	8	6	650	11	9	9	9	11	3	8	4	3	4
LGXI	13	10	20	6	7	7	9	4	441	7	7	5	4	4	3	1	3	3
LGVII	15	18	13	10	14	6	15	13	12	679	11	11	14	7	4	7	9	7
LGXVIII	5	2	7	3	9	3	5	1	3	3	366	4	1	3	4	1	2	2
LGXIII	7	5	8	7	5	6	3	5	2	9	64	532	6	3	4	2	3	5
LGX	6	8	4	8	9	1	9	5	7	5	13	6	823	5	5	6	12	5
LGXIX	2	0	0	0	0	0	0	0	1	0	0	0	61	0	0	0	1	0
LGXV	8	4	3	4	4	5	2	1	6	4	7	5	4	258	7	1	2	5
LGXXIII	0	0	0	0	0	0	0	0	0	0	1	0	1	14	0	0	0	0
LGXIV	9	8	4	6	3	6	5	2	8	3	2	5	4	1	380	3	5	1
LGXII	11	4	195	14	4	7	7	4	7	8	14	10	4	1	5	475	8	3
LGXXIV	0	0	0	0	0	0	0	0	0	0	0	0	0	0	0	10	0	1
LGXVI	3	6	5	3	3	2	8	4	5	3	91	2	4	2	0	2	318	3
LGXXI	0	1	1	1	0	0	0	0	1	0	0	0	0	0	0	0	26	0
LGXVII	3	5	4	8	3	2	5	3	4	0	3	2	4	4	2	1	6	303

Note:The table shows the unique or shared number of BB02 scaffolds (Adema et. al., 2017) matched to the linkage groups (LGs) of iM line (using Minimap 2) and to the LGs of an earlier study (derived from Table S4 of Tennessen et. al., 2017). The correlated matches of linkages groups (LG) from iM line (18 LGs in this study) and Tennessen et. al., 2017 (24 LGs) are highlighted by blue color.
