# Supplementary material for: Compatibility between snails and schistosomes: insights from new genetic resources, comparative genomics, and genetic mapping
Source: Commun Biol. 2022 Sep 9;5:940. doi: 10.1038/s42003-022-03844-5 (PMC9463173; doi:10.1038/s42003-022-03844-5)
Supplement: Supplementary file 2 — Description of Additional Supplementary Data [file 42003_2022_3844_MOESM2_ESM.pdf]

Description of Additional Supplementary Files

File name: Supplementary Data 1

Description: Coding sequences (exons) involved in structural variations (SVs) in iM line and iBS90 genomes

File Name: Supplementary Data 2

Description: Summary of phenotypes and reads mapping of F2 snails and their parents

File Name: Supplementary Data 3

Description: Genotypes of 4 parents and 116 F2 individuals at 966 SNP loci in the 18 linkage groups (LGs).

File Name: Supplementary Data 4

Description: Linkage groups and corresponding scaffolds in iM line and iBS90 genome assemblies.

File Name: Supplementary Data 5

Description: Differential SNPs detected in the QTL regions and their gene effect and mRNA products

File Name: Supplementary Data 6

Description: Genes associated with SNPs
