# Supplementary material for: Compatibility between snails and schistosomes: insights from new genetic resources, comparative genomics, and genetic mapping
Source: Commun Biol. 2022 Sep 9;5:940. doi: 10.1038/s42003-022-03844-5 (PMC9463173; doi:10.1038/s42003-022-03844-5)
Supplement: Supplementary file 9 — Reporting Summary [file 42003_2022_3844_MOESM9_ESM.pdf]

nature research

Corresponding author(s): Si-Ming Zhang

Last updated by author(s): July 17, 2022

Reporting Summary

Nature Research wishes to improve the reproducibility of the work that we publish. This form provides structure for consistency and transparency in reporting. For further information on Nature Research policies, see our Editorial Policies and the Editorial Policy Checklist.

Statistics

For all statistical analyses, confirm that the following items are present in the figure legend, table legend, main text, or Methods section.

n/a	Confirmed	
☐	☒	The exact sample size (*n*) for each experimental group/condition, given as a discrete number and unit of measurement
☐	☒	A statement on whether measurements were taken from distinct samples or whether the same sample was measured repeatedly
☐	☒	The statistical test(s) used AND whether they are one- or two-sided<br>*Only common tests should be described solely by name; describe more complex techniques in the Methods section.*
☐	☒	A description of all covariates tested
☐	☒	A description of any assumptions or corrections, such as tests of normality and adjustment for multiple comparisons
☐	☒	A full description of the statistical parameters including central tendency (e.g. means) or other basic estimates (e.g. regression coefficient) AND variation (e.g. standard deviation) or associated estimates of uncertainty (e.g. confidence intervals)
☐	☒	For null hypothesis testing, the test statistic (e.g. *F*, *t*, *r*) with confidence intervals, effect sizes, degrees of freedom and *P* value noted<br>*Give P values as exact values whenever suitable.*
☒	☐	For Bayesian analysis, information on the choice of priors and Markov chain Monte Carlo settings
☐	☒	For hierarchical and complex designs, identification of the appropriate level for tests and full reporting of outcomes
☒	☐	Estimates of effect sizes (e.g. Cohen's *d*, Pearson's *r*), indicating how they were calculated

Our web collection on statistics for biologists contains articles on many of the points above.

Software and code

Policy information about availability of computer code

Data collection	Software was not used for data collection because it is not required.
Data analysis	A large number of softwares or computational programs were used for data analysis and described in the paper. They include FastQC v. 0.11.9, Trimmomatic v0.36, Kraken v2.0.8, Canu 1.9, Pilon 1.23, SSPACE 3.0, Blobtools2, BLASTn program in NCBI-BLAST+ package 2.11.0, BUSCO 4.1.4, RepeatModeler 2.0.1, lnterProScan 5.45, RepeatMasker 4.0, EVidence Modeler 06/25/2012, tBLSATn, AUGUSTUS, CD-HIT-EST, EvidentialGene, BLASTp, Program to Assemble Spliced Alignments (PASA) pipeline, HISAT2, BRAKER 2.1.1, GeneMark-ET, minimap2 version 2.17-r941, SyRI, bedtools v2.29.2, R Statistical Computing Environment, R packages openxlsx 4.1.5, tidyverse 1.3.0, and ggplot2 3.3.2, Burrows-Wheeler Alignment tool (BWA), Samtools 1.2, CLC Genomics Workbench 12.0 (QIAGEN), QTL lciMapping version 4.2.53, PLINK, Haploview, MapChart, R/qtl, and R/ASMap.

For manuscripts utilizing custom algorithms or software that are central to the research but not yet described in published literature, software must be made available to editors and reviewers. We strongly encourage code deposition in a community repository (e.g. GitHub). See the Nature Research guidelines for submitting code & software for further information.

Data

Policy information about availability of data

All manuscripts must include a data availability statement. This statement should provide the following information, where applicable:

- Accession codes, unique identifiers, or web links for publicly available datasets
- A list of figures that have associated raw data
- A description of any restrictions on data availability

All raw sequences of the two genomes, ddRADseq reads, and assembled annotated genome sequences were submitted to one NCBI project with accession number PRJNA769727.

April 2020

Field-specific reporting

Please select the one below that is the best fit for your research. If you are not sure, read the appropriate sections before making your selection.

☒ Life sciences ☐ Behavioural & social sciences ☐ Ecological, evolutionary & environmental sciences

For a reference copy of the document with all sections, see nature.com/documents/nr-reporting-summary-flat.pdf

Life sciences study design

All studies must disclose on these points even when the disclosure is negative.

Sample size	126 phenotypied snail samples that were used for ddRADseq analysis have enough statistical power to identify QTL regions related to parasite resistance or body color. For generating high-quality genome sequence, it is better to use number of samples as less as possible. In our study, for each line, we use two homozygous snails (e.g., the two snails have identical genome).
Data exclusions	A total of 6 samples were dropped because the quality of reads is low
Replication	NA
Randomization	NA
Blinding	NA

Reporting for specific materials, systems and methods

We require information from authors about some types of materials, experimental systems and methods used in many studies. Here, indicate whether each material, system or method listed is relevant to your study. If you are not sure if a list item applies to your research, read the appropriate section before selecting a response.

Materials & experimental systems

n/a	Involved in the study	
☒	☐	Antibodies
☒	☐	Eukaryotic cell lines
☒	☐	Palaeontology and archaeology
☐	☒	Animals and other organisms
☒	☐	Human research participants
☒	☐	Clinical data
☒	☐	Dual use research of concern

Methods

n/a	Involved in the study	
☒	☐	ChIP-seq
☒	☐	Flow cytometry
☒	☐	MRI-based neuroimaging

Animals and other organisms

Policy information about studies involving animals; ARRIVE guidelines recommended for reporting animal research

Laboratory animals	Lab-rearing mice (Mus musculus) (3-4 month old) were used for production of schistosome eggs for infecting the snail Biomphalaria glabrata
Wild animals	The study did not involve wild animals.
Field-collected samples	The study did not involve samples collected from the field.
Ethics oversight	Details regarding ethics is provided in the section of Methods

Note that full information on the approval of the study protocol must also be provided in the manuscript.
